# In Situ Growth Intercalation Structure MXene@Anatase/Rutile TiO_2_ Ternary Heterojunction with Excellent Phosphoprotein Detection in Sweat

**DOI:** 10.3390/bios12100865

**Published:** 2022-10-12

**Authors:** Yuting Qiao, Xianrong Liu, Zhi Jia, Peng Zhang, Li Gao, Bingxin Liu, Lijuan Qiao, Lei Zhang

**Affiliations:** 1 School of Mechanical Engineering, Qinghai University, Xining 810016, China; 2 Research Center of Basic Medical Science, Medical College, Qinghai University, Xining 810016, China; 3 Department of Mechanical Engineering, University of Alaska Fairbanks, Fairbanks, AK 755905, USA

**Keywords:** sweat, phosphoprotein, MXene, anatae/rutile TiO_2_, intercalation structure, healthcare monitoring

## Abstract

Abnormal protein phosphorylation may relate to diseases such as Alzheimer’s, schizophrenia, and Parkinson’s. Therefore, the real-time detection of phosphoproteins in sweat was of great significance for the early knowledge, detection, and treatment of neurological diseases. In this work, anatase/rutile TiO_2_ was in situ grown on the MXene surface to constructing the intercalation structure MXene@anatase/rutile TiO_2_ ternary heterostructure as a sensing platform for detecting phosphoprotein in sweat. Here, the intercalation structure of MXene acted as electron and diffusion channels for phosphoproteins. The in situ grown anatase/rutile TiO_2_ with n-n-type heterostructure provided specific adsorption sites for the phosphoproteins. The determination of phosphoprotein covered concentrations in sweat, with linear range from 0.01 to 1 mg/mL, along with a low LOD of 1.52 μM. It is worth noting that, since the macromolecular phosphoprotein was adsorbed on the surface of the material, the electrochemical signal gradually decreased with the increase of phosphoprotein concentration. In addition, the active sites in the MXene@anatase/rutile TiO_2_ ternary heterojunction and synergistic effect of the heterojunction were verified by first-principle calculations to further realize the response to phosphoproteins. Additionally, the effective diffusion capacity and mobility of phosphoprotein molecules in the ternary heterojunction structure were studied by molecular dynamics simulation. Furthermore, the constructed sensing platform showed high selectivity, repeatability, reproducibility, and stability, and this newly developed sensor can detect for phosphoprotein in actual sweat samples. This satisfactory sensing strategy could be promoted to realize the noninvasive and continuous detection of sweat.

## 1. Introduction

Sweat is a biological sample that allows for real-time continuous monitoring, which has been pushed into the focus of research, due to its own availability and potential for continuous collection, as a potential source of real-time personal biomonitoring. Sweat contains various vital substances, such as electrolytes, metabolites, nutrients, and protein molecules [1]. Therefore, realizing the monitoring of metabolites in sweat is of unique and important value to the prevention, diagnosis, and treatment of high-incidence diseases. 

Human-excreted sweat contains relatively low concentrations of protein (about 0.2–1.02 mg/mL) [2,3]. Proteins in the human body need to undergo post-translational modifications to further exert their corresponding biological functions. Protein phosphorylation was the most common way of regulating life activities and one of the most important post-translational modifications. As a rapidly reversible means to further regulated protein activity and signal transduction, it was involved in the coordination of essentially all cellular processes [4]. However, abnormal protein phosphorylation was associated with the occurrence of various diseases, such as cancer, inflammatory diseases, diabetes, infectious diseases, cardiovascular diseases, etc. [5,6,7,8,9,10]. Therefore, the non-invasive real-time monitoring of phosphoproteins in sweat through electrochemical sensing platforms was of great significance for human disease prevention. However, the detection of phosphoproteins based on human sweat is rarely reported.

It is worth mentioning that the protein molecules have difficulty achieving direct heterogeneous electron transfer reactions on the electrode surface, and the reversibility was also poor. This could be attributed to the non-uniform distribution of the surface charges of the protein molecules and denaturing adsorption on the electrode surface, which further led to a low rate of the mass transfer process of the protein, which inhibited the electron transfer process between the electrode and protein [11,12]. Therefore, improving the direct electron transfer of proteins in biosensors remains a challenging work. Up to now, some studies have further achieved direct electron transfer from proteins by modifying dielectric material, such as metal oxides, alloy nanoparticles, and carbon materials [13,14,15].

In recent years, two-dimensional (2D) transition metal carbides and/or carbonitrides materials, such as MXenes, have attracted extensive attention, due to their excellent properties, such as electronic transport properties, large specific surface, good biocompatibility, hydrophilicity, and chemical diversity [16]. However, the enrichment efficiency for phosphoproteins was relatively poor, due to the lack of affinity-specific active sites for MXenes [17].

The TiO_2_-based metal oxide affinity chromatography (MOAC) had excellent affinity and binding selectivity to phosphoproteins, and it was a specific material in the research of phosphoproteomics [18]. Compared with traditional separation media, TiO_2_ had higher chemical stability, rigidity, and better zwitterion exchange performance [19]. Thus, TiO_2_ had further applications in the detection of phosphoproteins, based on the interaction of the Lewis acid-base properties of TiO_2_ with phosphate groups [20,21].

Herein, the intercalation structure heterostructuring MXene@anatase/rutile TiO_2_ was facile prepared through a one-step calcination process and employed as sensing materials for the electrochemical detection of phosphoprotein in sweat. We synthesized the sensing material in situ by calcination, under the protection of Ar atmosphere; the in situ calcination generated anatase/rutile TiO_2_ nanoparticles in the MXene-layered structure with good electrical conductivity and formed a composite, which provided abundant active sites, hierarchical mass transfer channels, and sensing performance. Furthermore, the structure–activity relationship between the MXene@anatase/rutile TiO_2_ ternary heterojunction and phosphoprotein was determined by first-principle calculations. Molecular dynamics simulations were used to verify the migration ability and spreadability of phosphoprotein molecules in the ternary heterojunction. More importantly, we have developed a facile electrochemical strategy for the real-time monitoring of phosphoproteins in sweat, which could further be widely applied in the health monitoring field. 

## 2. Materials and Methods

### 2.1. Materials Preparation

Ti_3_AlC_2_ (98.0%), hydrofluoric acid (HF, 99.0%), potassium ferricyanide (K_3_[Fe(CN)_6_], 99.5%), potassium ferrocyanide (K_4_[Fe(CN)_6_], 98.0%), potassium chloride (KCl, 99.5%), titanium dioxide (TiO_2_, rutile, anatase), bovine serum albumin (BSA), Nafion 117 solution (5%), β-Casein, α-lactalbumin, and β-lactoglobulin were purchased from Aladdin (Shanghai, China). Absolute ethanol was purchased from Sinopharm Chemical Reagent Co. (Shanghai, China). Artificial sweat was purchased from Dongguan Xinheng Technology (pH = 5.0, 6.0, 7.0, 8.0, and 9.0, Guangdong, China). All chemical reagents were analytical-grade and directly used without further refinement.

### 2.2. Characterizations

The crystallinity and phase structure of materials were measured by X-ray diffraction (Bruker AXS Company, Karls ruhe, Germany) in the range of 5–80° by a radiation source (λ = 1.5418 Å). The group composition of the sensing materials was collected by Fourier transform infrared spectrometer (Thermofisher Nicolet 6700, Waltham, MA, USA), with KBr as reference. The microstructure of the sensing materials was observed by scanning electron microscope (JSM-7900F, JEOL, Tokyo, Japan) and transmission electron microscope (EM-2100FE, JEOL, Tokyo, Japan), which were equipped with energy-dispersive spectroscopy (EDS). The Raman spectra were studied by Horiba LabRAM Odyssey Raman microscope (LW-10, Lille, France) at 532 nm excitation laser. The surface composition was examined by X-ray photoelectron spectroscopy spectra (AXIS-ultra DLD, Manchester, England) at 284.8 eV for calibration. The optical properties of the sensing material were detected with an ultraviolet spectrophotometer (UV-4802S, Zhengzhou, China), and the band gap value was obtained by the photoelectric equation. Atomic force microscopy (AFM) images of the sensing materials were scanned in tapping mode by atomic force microscope (Dimension® Icon™, Karls ruhe, Germany). The thermal stability was carried out with synchronous thermal analyzer (STA449, Selb, Germany) at 10 °C/min in the air. BET specific surface area and pore size were measured by automatic specific surface and pore size distribution analyzer (BSD660, Beijing, China). The zeta potential of sensing materials was measured by the malvern nanoparticle size analyzer (ZS-90, Melvin, UK). Cyclic voltammetry (CV), electrochemical impedance spectroscopy (EIS) measurements, differential pulse voltammetry (DPV), and chronoamperometry (CC) were taken by electrochemistry workstation (CHI660-E, Shanghai, China) with a standard three-electrode system. The construction of the sensor array was completed by a desktop glue dispenser (KW-4A, Hebei, China), and the real-time monitoring test was completed by a portable electrochemical workstation (MEC-VS1, Guangdong, China).

### 2.3. Synthesis of MXene

The preparation method of MXene was slightly modified, according to previous work [22,23]. Typically, 3 g MAX phase precursor (Ti_3_AlC_2_) powder were gradually added into reactor for selective etching with 60 mL of 40% HF. The mixed solution was kept stirred at 25 °C for 24 h, and then the resulting MXene suspension was then washed with deionized water (DW) and ethanol by centrifugation at 5000 rpm, until the pH value of the supernatant was greater than 6. Finally, the precipitate was dried in a vacuum oven at 80 °C for 12 h to obtain accordion-shaped MXene.

### 2.4. Synthesis of MXene@TiO_2_

The 2 g accordion-shaped MXene samples were taken and placed in a tube furnace, and Ar was passed through to remove O_2_ for 30 min. Then, in an Ar atmosphere, the temperature was increased at a heating rate of 5 °C/min, and the samples were calcined at 200, 400, 600, and 800 °C for 2 h to obtain MXene@TiO_2_ samples with different morphologies. According to the different heating rates, the samples were labeled as MT-200 °C, MT-400 °C, MT-600 °C, and MT-800 °C, respectively.

### 2.5. Electrochemical Measurement

The sensing performance was tested with an Ag/AgCl electrode, platinum wire (Pt) electrode, and glassy carbon electrode (GCE) as reference, counter, and working electrodes with surface modification, supplemented by 5 mM K_3_[Fe(CN)_6_]/K_4_[Fe(CN)_6_] and 0.1 M KCl, 0.1 mM K_3_[Fe(CN)_6_] and 1 M KCl, or artificial sweat as the electrolyte solutions for electrochemical detection.

Specifically, bare GCE with a diameter of 3 mm were sequentially polished with 1, 0.3, and 0.05 μm alumina powder, rinsed thoroughly with DW and ethanol, and dried. Before electrochemical testing, the three electrodes were subjected to CV cycling in the electrolyte at a voltage of −0.4 to 0.6 V and scan rate of 100 mV/s. The working electrode could be preconditioned, until a stable redox signal appeared. A total of 5 mg of the composite powder was dissolved into 1 mL mixed solution (500 μL DW, 470 μL ethanol, and 30 μL Nafion) to uniformly dispersed by sonication. Then, the 8 μL as-prepared solution was dropped uniformly onto bare GCE and left to air-dry naturally. N_2_ was used to remove O_2_ interference prior to all electrochemical tests.

### 2.6. Preparation of Sensing Array Construction

First, the three electrodes of the PI flexible substrate were ultrasonically cleaned in DW for 30 minutes, then cleaned with ethanol and air-dried naturally. Subsequently, 25 mg of MT-600 °C composite were taken to disperse in 5 mL of solution (V_DW_:V_Ethanol_:V_Nafion_ = 10:9:1) for ultrasonic dispersion. After that, spin-coating was performed on the PI flexible substrate at room temperature at 2000 rpm for 60 s, and it was naturally dried to ensure the completion of the sensor array construction.

## 3. Results and Discussion

### 3.1. Characterization of the Sensing Materials

**Figure 1:**
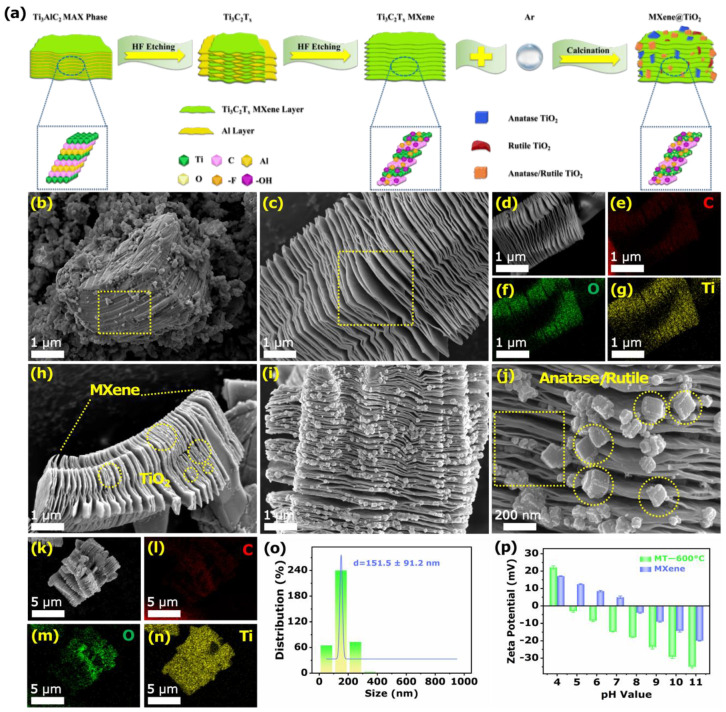
(**a**) Schematic illustration of the synthesis of MXene@TiO_2_ composites. SEM images of (**b**) Ti_3_AlC_2_, (**c**,**d**) MXene, and mappings of (**e**) C, (**f**) O, and (**g**) Ti elements. SEM images of (**h**–**k**) MT-600 °C, under different angles and mappings of (**l**) C, (**m**) O, and (**n**) Ti elements. (**o**) Particle size distribution histogram. (**p**) Zeta potential of MXene and MT-600°C with different pH value.

Commonly, MXene@TiO_2_ composites used in different research fields were usually synthesized by tedious methods, such as hydrothermal method and co-precipitation [24,25,26]. In contrast, MXene@TiO_2_ composites with intercalation structures and crystal were prepared by a facile one-step calcination under Ar gas protection. The synthesis process of the intercalation structure MXene@TiO_2_ is shown in Figure 1a. The morphological transition of the sensing material was investigated by scanning electron microscopy (SEM). The bulk Ti_3_AlC_2_ had a layer-by-layer texture structure, and the dense morphology between layers was shown in Figure 1b. Subsequently, Al atoms in Ti_3_AlC_2_ were selectively etched by HF; a large amount of heat was generated, and a large amount of H_2_ was released during the reaction. The MXene formed by internal expansion showed a typical layered exfoliation two-dimensional morphology (Figure 1c) [27]. Figure 1d showed that the elemental mapping images corresponding to the MXene structure included the uniform distribution of C, O, and Ti elements (Figure 1c–g). The intact Ti atoms at the surface edge of MXene could be used as nucleation sites and rich groups, such as -O and -OH, at the surface end to construct stable TiO_2_, which was formed a heterojunction structure to facilitate electron transfer [28,29]. The smooth surface of MXene became rougher, anchored inside the multilayer, and the surface was tightly surrounded to form a unique sandwich structure. Simultaneously, a potential barrier was formed between the metallic phase MXene and semiconducting TiO_2_ to further self-assemble and form the MXene@TiO_2_ hybrid nanostructure [30]. This interface structure was beneficial for facilitating the transfer of electrons and providing more adsorption sites for phosphoproteins in the sensing response [31].

In addition, the structure and content of TiO_2_ changed simultaneously with calcination and oxidation as the temperature increased from 200 to 800 °C. Firstly, many TiO_2_ nanosheets grew in situ on the surface and interior of the multilayer as the temperature increased to 200 and 400 °C, and further elemental mapping demonstrated the relatively uniform coverage of TiO_2_ nanosheets on the MXene substrate (Figures S1 and S2). As higher calcination temperatures led to more energy excess, further enhanced atomic diffusion led to rapid growth of TiO_2_ gained. When the temperature was further increased to 600 °C (Figure 1h), it was obvious that the edges of the TiO_2_ nanosheets inside and between the layers of MXene gradually sharpened and became uneven (Figure 1i,j). The element map further showed the relatively uniform degree of C, O, and Ti elements of MT-600 °C (Figure 1k–n). The average grain size of TiO_2_ nanoparticles in MT-600 °C was 151.5 ± 91.2 nm by particle size statistics (Figure 1o). It is worth noting that the accordion-like structure of MXene was well-preserved at 200 (Figure S1a), 400 (Figure S2a), and 600 °C (Figure 1h). Furthermore, when the calcination temperature reached 800 °C, the layered structure apparently belonging to MXene was collapsed. At this moment, a certain amount of TiO_2_ was further transformed into TiO_2_ nanorods interspersed with each other (Figure S3a). It was further observed that the MXene nanosheets were almost completely transformed into dense and irregular accumulations of massive TiO_2_ nanoparticles. The same elemental mapping distribution showed a uniform distribution of C, O, and Ti components on the sample surface (Figure S3c–f). 

In addition, the experiments further confirmed the simultaneous decrease of oxidized functional groups in MT-600 °C, with the increase of the oxidation degree of MXene, by measuring the zeta potential. The reported data was derived from the average of three zeta potential tests. According to the change trend of zeta potential in Figure 1p, it could be seen that the zeta potential of MXene and MT-600 °C were 4.85 and 7.65, respectively, both of which were positive, and the electronegativity increased with the increase of pH value. However, the electronegativity of MT-600 °C was more negative than that of MXene; this result indicated that the degree of oxidation increases with the degree of alkalinity in aqueous solution. More basic groups were adsorbed on the surface of MT-600 °C, which resulted in a higher degree of oxidation and further formation of TiO_2_, thus providing more adsorption sites for attracting phosphoprotein molecules [32].

**Figure 2:**
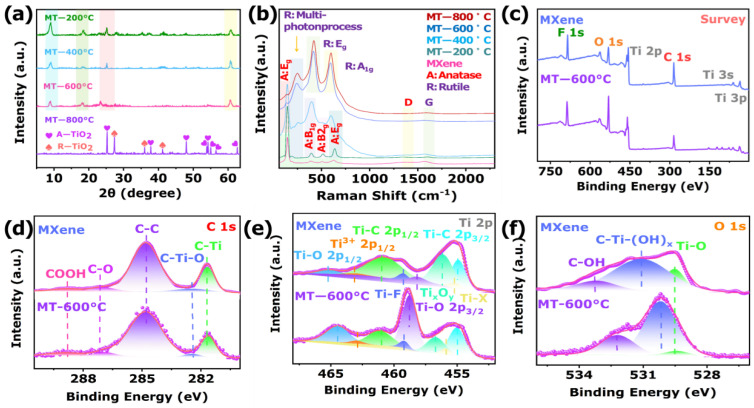
XRD patterns of (**a**) Ti_3_AlC_2_, MXene, MT-200 °C, MT-400 °C, MT-600 °C, MT-800 °C. (**b**) Raman spectra of MXene, MT-200 °C, MT-400 °C, MT-600 °C, MT-800 °C. XPS spectra of MXene and MT-600 °C: (**c**) survey, (**d**) C 1s, (**e**) O 1s, and (**f**) Ti 2p.

The phase and crystal structure of sensing materials were characterized by X-ray diffraction data (XRD). As illustrated in Figure S4, three strongest peaks at 9.54°, 37.16°, and 50.62° corresponded to (002), (104), and (108) crystal planes of Ti_3_AlC_2_ (JCPDS PDF#52-0875), respectively [33]. In addition, the diffraction pattern of the MXene demonstrated 8.7°, 16.54°, 28.4°, and 61.46° intense peaks, which corresponded to (002), (006), (008), and (110) crystal planes (JCPDS PDF#43-1456), respectively. This indicated that the MXene material had a two-dimensional layered structure [34]. It is worth noting that the XRD pattern of MXene showed that the (104) diffraction peak attributed to Ti_3_AlC_2_ disappeared after HF chemical etching, which indicated that the Al atoms in Ti_3_AlC_2_ were removed by etching. In addition, the (002) characteristic peak of Ti_3_AlC_2_ was significantly left-shifted after HF etching, which indicated that the interplanar spacing along the C-axis increased significantly [35]. Therefore, the shift of the (002) characteristic peak and disappearance of the (104) characteristic peak indicated that the crystal structure of Ti_3_AlC_2_ has changed, and 2D MXene materials were successfully prepared. Compared with an untreated MXene, the diffraction peaks of different crystal structures of TiO_2_ were observed after the calcining oxidation of MXene (Figure 2a). Specifically, the main peak at 8.7° belongs to MXene after calcination decreased significantly with the increase of temperature from 200 to 800 °C. This indicated that the layered structure of MXene was still maintained after high temperature calcination, but the MXene was partially transformed. After calcination and oxidation from 200 to 400 °C, some sharp diffraction peaks were detected at 25.3°, 37.8°, 48.0°, 53.86°, 54.2°, 55.0°, and 62.6°, which were attributed to (101), (004), (200), (105), (211), (201), and (204) planes of anatase TiO_2_ (JCPDS PDF#21-1272), respectively. This corresponded to the -OH functional groups on the etched MXene surface and Ti element form TiO_2_ [36]. As the temperature increased to 800 °C, new diffraction peaks were detected at 27.4°, 36.0°, and 41.2°, belonging to (110), (101), and (111) planes of the rutile TiO_2_ (JCPDS PDF#21-1276), respectively [37]. This indicated that the increase of temperature was also accompanied by the transformation of anatase phase to rutile phase. Therefore, the XRD demonstrated that the calcination temperature affects the heterojunction of in situ growth of TiO_2_ of different crystal types on the surface and interior of MXene [38]. 

The Raman spectrum was further used to elucidate the crystalline phase of sensing materials (Figure 2b). Two peaks at 156 and 196 cm^−1^ of MXene belonged to the in-plane and stretching vibrations of surface Ti atoms of A_1g_. Additionally, the peak at 626 cm^−1^ of MXene was assigned to the stretching vibration of surface C atoms of A_1g_. In addition, the 297, 384, and 503 cm^−1^ peaks corresponded to the in-plane vibrations (E_g_) of the H, O, and C atoms [39]. Therefore, the vibrational modes of MXene suggested that Ti_3_AlC_2_ was successfully etched. Additionally, the Raman spectra of the MXene@TiO_2_ under different temperature showed that the peaks at 139, 395, 510, and 634 cm^−^^1^ corresponded to typical E_g_, B_1g_, B_2g_, and E_g_ Raman vibrations of anatase TiO_2_, respectively. Moreover, the Raman peaks at 243, 414, and 596 cm^−^^1^ were assigned to multi-photon process E_g_ and A_1g_ vibrations of rutile TiO_2_ in MT-800 °C, respectively [40]. However, the Raman peak of MT-600 °C was between the peak of anatase and rutile phases. This result indicated that the anatase and rutile phases of TiO_2_ coexisted in MT-600°C [41]. In addition, it was found that the intensity of anatase peak decreased gradually, and rutile peak gradually increased with the increase of calcination temperature, as well as due to sub-stoichiometric Ti_x_O_y_ and size effects of TiO_2_, which led to the E_g_ band of rutile TiO_2_ left shifts [42,43]. Furthermore, the broad peaks between 1000 and 1800 cm^−1^ belonged to the characteristic peaks of the D- and G-band modes of graphitic carbon. It is worth noting that the Raman peaks at 1330 and 1590 cm^−1^ of MT-600 °C belonged to the D-band vibrational peak of the sp^3^-hybridized structure and G-band vibrational peak of the sp^2^-hybridized structure of the surface carbon, respectively. The D/G band ratios in the carbon peaks of MT-600 °C and MXene were about 0.830 and 0.882, respectively. These results showed that the content of graphitized carbon in the MT-600 °C, after MXene calcination, increased and assembled to TiO_2_ to enhance the conductivity [44].

To investigate the elemental chemical composition and chemical state of the sensing materials, the X-ray photoelectron spectroscopy (XPS) measurements were performed. Full spectrum analysis of MXene and MT-600 °C are shown in Figure 2c, and the MXene and MT-600 °C were composed of Ti, O, C, and F elements (Table S1). In addition, the F element on the MXene and MT-600 °C was attributed to the physical adsorption of F ions in the HF solution. Figure 2d shows the C 1s spectra of MXene and MT-600 °C, and the binding bonds and corresponding binding energy are shown in Table S2. The intensity peak of the C-Ti (281.68 eV) in MT-600 °C was gradually decreased with the prolongation of calcination duration, which was due to the gradual oxidation of MXene and conversion to TiO_2_ [45,46,47,48]. The O 1s XPS spectra of MXene and MT-600 °C are shown in Figure 2e, and the specific peak intensity information is shown in Table S3. Particularly, the proportion of O element in MXene and MT-600 °C were 18.24% and 32.43%, respectively. The elemental analyses performed by XPS prove that the O atoms in MT-600 °C have observably increased (Table S1). However, the bond of C-Ti-(OH)_x_ accounted for 89.69% in the O1s, which proved that a large amount of -OH was adsorbed on the surface of the MXene. After oxidation, the proportion of C-Ti-(OH)_x_ in MT-600 °C decreased to 65.87%. This phenomenon indicated that -OH in MXene was gradually consumed, and TiO_2_ was formed during the calcination process. It also proved that many -OH groups were terminated on the MXene surface of the MT-600 °C composite [49,50,51]. The core doublets in the high-resolution Ti 2p spectrum (Figure 2f) of MXene and MT-600 °C were Ti 2p_3/2_ and Ti 2p_1/2_ peaks (Table S4), which were corresponded to the spin-orbit splitting states of photoelectrons in Ti^3+^/Ti^4+^, respectively [52,53]. It is worth noting that the continuation of the calcination reaction in MT-600 °C led to a decrease in the peak intensity of Ti-C bonds, which corresponded to the further conversion of most MXene into semiconducting TiO_2_ [54,55,56,57]. This was consistent with the phenomenon observed by C 1s spectrum. The above results clearly showed that MT-600 °C has been successfully prepared.

**Figure 3:**
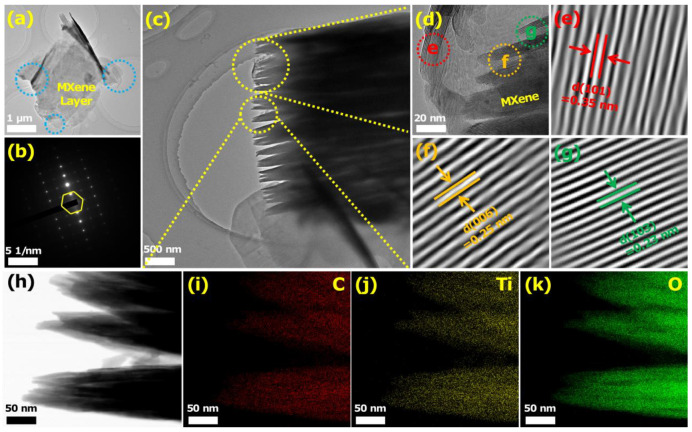
(**a**) TEM of MXene and (**b**) the corresponding SAED patterns. (**c**) TEM of MXene, (**d**) the corresponding HRTEM images, and the selected area (**e**–**g**) spacing images of (**e**) (101) plane, (**f**) (006) plane, and (**g**) (103) plane of MXene. (**h**) Bright-field scanning TEM image and EDX elemental mapping of the (**i**) C, (**j**) Ti, and (**k**) O.

Further structural elucidation of sensing materials was accomplished by transmission electron microscope (TEM). As can be seen in Figure 3a, the MXene showed a well-stacked hierarchical structure. Further, it could be found that the MXene sheets layer were smooth and clear, and no traces of secondary materials were found. Furthermore, the SAED pattern, as shown in Figure 3b, confirmed the hexagonal symmetric crystal system of the MXene structure, which indicated that it retained the crystallinity and symmetry of the MAX phase Ti_3_AlC_2_ [58]. However, the obvious multilayer structure of MXene was clearly shown in Figure 3c. The further HRTEM image (Figure 3d) showed the typical lattice fringes of MXene, where the three lattice spacings of 0.35, 0.25, and 0.23 nm could specifically corresponded to the (101), (006), and (103) crystal planes (Figure 3e–g). The elemental mapping of 2D layered MXenes indicated uniform distribution and presence of C, O, and Ti elements (Figure 3h–j). The above results indicated that 2D MXene were successfully prepared. 

**Figure 4:**
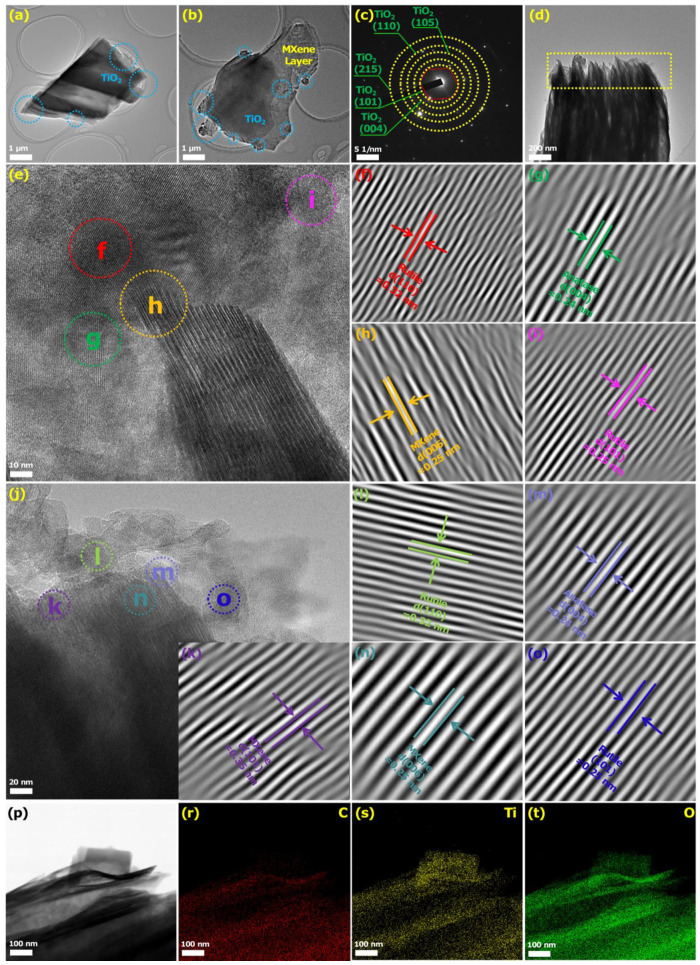
(**a**,**b**) TEM of MT-600 °C and (**c**) the corresponding SAED patterns. (**d**) TEM of MT-600 °C and the corresponding HRTEM images of (**e**–**j**). The spacing images of TiO_2_ (anatase, rutile) and MXene planes of HRTEM images of (**e**,**j**) with the selected areas (**f**–**i**) and (**k**–**o**), respectively. (**p**) Bright-field scanning TEM image and EDX elemental mapping of the (**r**) C, (**s**) O, and (**t**) Ti.

Figure 4 further shows the microstructure of the MT-600 °C. Figure 4a shows that the lamellar structure of MXene became rough and layered with secondary biomass attached. Similarly, it was found that many TiO_2_ nanoparticles formed by in situ oxidation were uniformly distributed in the edge and inner structure of the MXene sheet from Figure 4b. The lamellar structure of MXene was still well-maintained. The corresponding SAED pattern (Figure 4c) was shown to consist of a set of hexagonal lattice diffraction crystal systems and series of concentric diffraction rings, which corresponded to the typical 2D MXene structure and anatase/rutile phase of TiO_2_ nanoparticles, respectively [59,60]. Figure 4d showed the obvious layered structure of MXene, while the attachment of some TiO_2_ nanoparticles derivatives could be found, in comparison with Figure 3c. The growth of TiO_2_ nanoparticles between the MXene layers increased the specific surface area and interlayer spacing of the composite, which was beneficial for providing more phosphoprotein detection sites. Figure 4e,j were further HRTEM images of the composite, further corroborating the crystal structure phase of the composite. Among them, the lattice fringes of 0.32, 0.24, 0.25, and 0.25 nm in the selected region of Figure 4f–i in Figure 4e corresponded to the rutile phase (110), anatase phase (004), MXene (006), and rutile phase (101) crystal planes, respectively. Similarly, the lattice fringes of 0.35, 0.32, 0.24, 0.25, and 0.25 nm in the selected region of Figure 4k–o in Figure 4j, which corresponded to MXene (101), rutile phase (110), anatase phase (004), MXene (006), and rutile phase (101) crystal planes, respectively. The above lattice fringes indicated that anatase/rutile TiO_2_ nanoparticles have been successfully grown in situ on MXene. This also proved the formation of a heterojunction structure at the interface between MXene and anatase/rutile TiO_2_. Furthermore, the Figure S5 showed the in situ formation of anatase/rutile TiO_2_ nanoparticles on MXene sheets. The elemental mapping of the composites also showed uniform distribution and presence of C, O, and Ti elements (Figure 4p,r–t).

In addition, further characterization by Fourier transform infrared spectroscopy (FT-IR) showed that the MT-600 °C contained vibrational absorption peaks belonging to MXene and TiO_2_ (Figure S6). Furthermore, the physicochemical parameters of MT-600 °C were investigated by N_2_ adsorption-desorption isotherms, and the results showed that its specific surface area, pore volume, and pore size were 12.7380 m^2^/g, 0.042259 cm^3^/g, and 14.3820 nm, respectively (Figure S7). Additionally, the synthesized MT-600 °C sensing material had a good thermal stability at room temperature (Figure S8). Notably, the UV–Vis diffuse reflectance spectrum (DRS) of MT-600 °C showed strong UV absorption and broad characteristic peaks from 750 to 850 nm. Such interval windows were widely used in biomedical applications (Figure S9). Furthermore, further investigation of the morphological information of MT-600 °C by atomic force microscopy (AFM) indicated that the presence of TiO_2_ nanoparticles increased the interlayer spacing of MXene (Figure S10).

### 3.2. Phosphoprotein Sensing

**Figure 5:**
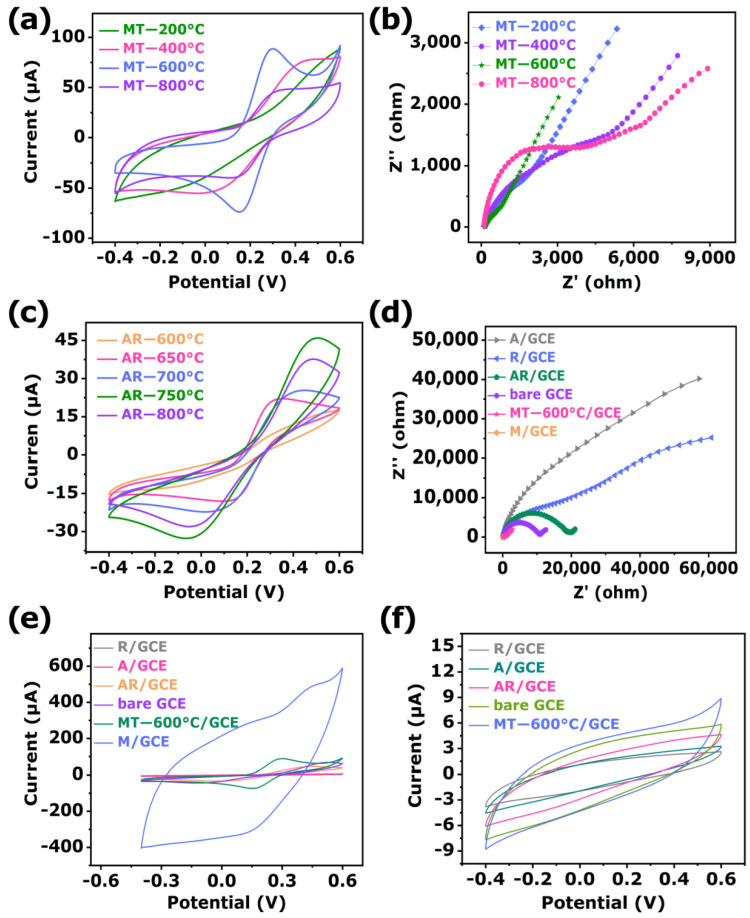
(**a**) CV and (**b**) EIS of the MT-200, MT-400, MT-600, and MT-800 °C in 5 mM [Fe(CN)_6_]^3−^/^4−^ solution containing 0.1 M KCl. (**c**) CV of the AR-600, AR-650, AR-700, AR-750, and AR-800 °C in 5 mM [Fe(CN)_6_]^3−^/^4−^ solution containing 0.1 M KCl. (**d**) EIS and (**e**) CV of the bare GCE, A/GCE, R/GCE, AR/GCE, M/GCE, and MT-600°C/GCE in 5 mM [Fe(CN)_6_]^3−^/^4−^ solution containing 0.1 M KCl. (**f**) CV of the bare GCE, A/GCE, R/GCE, AR/GCE, and MT-600 °C/GCE in artificial sweat (pH = 7.0) containing 1 mg/mL β-Casein.

Cyclic voltammetry (CV) was used to study the effect of phosphoproteins on the sensor current intensity under different electrochemical test system conditions. The CV images of the sensing materials at different temperatures were shown in Figure 5a. Obviously, it could be seen that the sensing materials exhibit typical redox peaks. Among them, the redox peak current intensity could be sorted as MT-600 °C > MT-200 °C > MT-400 °C > MT-800 °C. Moreover, electrochemical impedance spectroscopy (EIS) was employed to evaluate the capability of charge transfer resistance (Rct) on the surface of the modified electrode. Typically, the diameter of the semicircular portion of the EIS spectrum represented the Rct [61]. As shown in Figure 5b, the sensing materials of MT-600 °C (465 Ω) obtained a smaller semicircle diameter, and the Rct value was significantly smaller than that of the MT-200 (1531 Ω), MT-400 (3648 Ω), and MT-800 °C (3925 Ω) sensing materials. This means that MT-600°C had higher conductivity, which attributed to the heterojunction formed by anatase/rutile TiO_2_, and MXene formed by calcination at 600 °C could better promote the transfer of electrons. This result appeared to be consistent with the CV response. Therefore, the electrochemical response of MT-600 °C might be attributed to the existence of ternary heterojunction between MXene and anatase/rutile TiO_2_.

To demonstrate the important role of anatase/rutile TiO_2_ heterojunction in MT-600 °C, the anatase/rutile TiO_2_ was constructed by in situ calcined anatase TiO_2_ at 600, 650, 700, 750, and 800 °C (Figures S11 and S12). As shown in Figure 5c, the CV current intensity responses of pure anatase phase TiO_2_ calcined at 600, 650, 700, 750, and 800 °C were investigated. It could be clearly seen that the heterojunctions formed by sintering at different temperatures exhibit different current intensities. This indicated that the in situ growth at the two-phase interface formed an n-n-type heterojunction between the anatase and rutile phases, which further promoted the rapid charge transfer [62]. 

The EIS plots of different modified sensing materials are shown in Figure 5d. The Rct values of bare GCE, R/GCE, A/GCE, AR/GCE, M/GCE, and MT-600°C/GCE were estimated to be 10692, 39883, 31999, 19497, 35.5, and 465 Ω, respectively. Obviously, because of the high conductivity of MXene [63], the Rct of M/GCE was much smaller than that of other modified GCE. After the in situ growth of anatase/rutile TiO_2_ on MXene, the Rct of MT-600 °C/GCE became larger, compared with M/GCE. However, as a comparison experiment, AR/GCE had significantly lower Rct than A/GCE and R/GCE, but greater than that of MT-600 °C/GCE, which was caused by the formation of heterojunctions between MXene and anatase/rutile TiO_2_ to accelerate the charge transfer [64]. Interestingly, the Rct of the AR/GCE was significantly lower than that of the A/GCE and R/GCE, which could be attributed to the formation of heterojunction between the anatase and rutile from the anatase transformation. Moreover, the in situ growth of anatase/rutile TiO_2_ on MXene dramatically decreased the Rct of A/GCE, R/GCE, and AR/GCE, which illustrated that MXene improved the conductivity of the composite and facilitates the transfer of electrons. The construction of the modified electrodes was also studied by CV range from −0.4 to 0.6 V at a scan rate of 100 mV/s with 5 mM [Fe(CN)_6_]^3−^/^4−^ solution containing 0.1 M KCl as the redox probe. As presented in Figure 5e and Figure S13, a pair of typical redox characteristic peaks appeared at bare GCE. After being modified with MXene on bare GCE, the redox peak currents increased remarkably, which depended on excellent electronic conductivity, excellent electrocatalytic activity, and a huge active area for MXene to accelerate the electron transfer [62]. However, the peak current decreased by modified MT-600 °C on bare GCE than M/GCE, which is ascribed to the poor conductive semiconductor material anatase/rutile TiO_2_ [65]. Nevertheless, the MT-600 °C/GCE peak current was obviously higher than that of A/GCE, R/GCE, and AR/GCE. The remarkable enhancement could be assigned to the synergistic effect of MXene and anatase/rutile TiO_2_. Additionally, it was found that the currents of A/GCE and R/GCE were significantly lower than that of AR/GCE, which was consistent with the EIS results.

The CV behaviors of β-Casein on the modified electrodes were studied in artificial sweat (pH = 7.0). Figure 5f and Figure S14 depicted the CV responses of the bare GCE, A/GCE, R/GCE, AR/GCE, M/GCE, and MT-600 °C/GCE in artificial sweat (pH = 7.0) after incubation in 1 mg/mL β-Casein. As shown, a weak redox peak occurs at bare GCE, while a pair of strong obvious redox peaks were observed at M/GCE, which was owed to the prominent conductivity of MXene. Furthermore, MT-600 °C/GCE exhibited a much higher redox peak current than that of A/GCE, R/GCE, and AR/GCE. The conspicuous enhancement was attributed to the synergetic combination of anatase/rutile TiO_2_ and MXene, which enabled the modified electrode to have a large specific surface area, good conductivity, and excellent electrochemical activity. These results proved the superiority of MT-600 °C for phosphoprotein detection.

**Figure 6:**
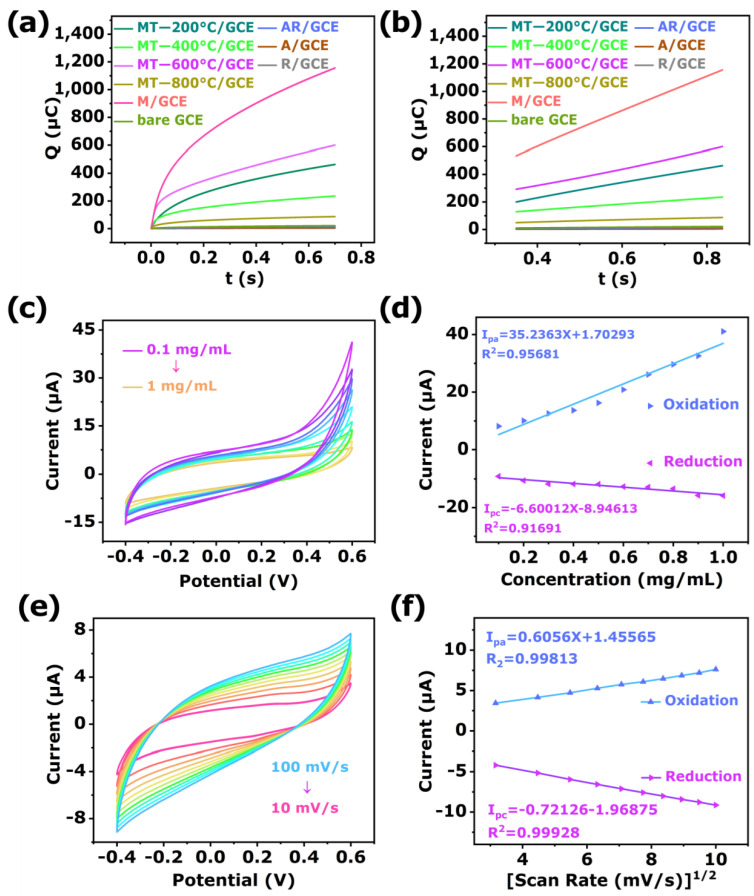
Plot of (**a**) Q-t and (**b**) Q-t^1/2^ curves of the bare GCE, A/GCE, R/GCE, AR/GCE, M/GCE, and MT-600 °C/GCE in 0.1 mM K_3_[Fe(CN)_6_] solution containing 1 M KCl. (**c**) CV of different concentrations (from 0.1 to 1 mg/mL) of β-Casein on MT-600 °C/GCE in artificial sweat (pH = 7.0) and (**d**) linear relationship between the redox peak currents and the concentration of β-Casein. (**e**) CV of MT-600 °C/GCE in artificial sweat (pH = 7.0) containing 1 mg/mL β-Casein with different scan rates (from 10 to 100 mV/s) and (**f**) linear relationship between the redox peaks currents and scan rate square root.

The electroactive specific surface areas of the bare GCE, A/GCE, R/GCE, AR/GCE, M/GCE, MT-200 °C/GCE, MT-400 °C/GCE, MT-600 °C/GCE, and MT-800 °C/GCE were investigated via chronocoulometry (Figure 6a,b and Figure S15a,b) and calculated by Anson Equation (1) [66]:

(1) $${Q(t)=(2\mathrm{nFAcD}}^{1/2}t^{1/2}{)/\pi }^{1/2}{+Q}_{\mathrm{dl}}{+Q}_{\mathrm{ads}}$$

Herein, n served as the number of electrons transferred. F stood for the Faraday’s constant (96,485 C/mol). A referred to the effective area of the electrode (cm^2^). c represented the substrate concentration (0.1 mol/cm^3^). D acted as diffusion coefficient (7.6 × 10^−6^ cm^2^/s). Additionally, the double layer and Faradic charges were denoted by Q_dl_ and Q_ads_, respectively. 

As shown, the linearization equations between Q and t^1/2^ were: bare GCE: Q (10^−6^C) = 10.75 + 21.65 t^1/2^ (R^2^ = 0.999); A/GCE: Q (10^−6^C) = 3.137 + 7.1 t^1/2^ (R^2^ = 0.999); R/GCE: Q (10^−6^C) = 1.881 + 3.2 t^1/2^ (R^2^ = 0.999); AR/GCE: Q (10^−6^C) = 6.606 + 14.86 t^1/2^ (R^2^ = 0.999); M/GCE: Q (10^−6^C) = 534.5 + 1113.2 t^1/2^ (R^2^ = 0.999); MT-200 °C/GCE: Q (10^−6^C) = 4.653 + 441 t^1/2^ (R^2^ = 0.999); MT-400 °C/GCE: Q (10^−6^C) = 2.5213 + 441 t^1/2^ (R^2^ = 0.999); MT-600 °C/GCE: Q (10^−6^C) = 5.9564 + 441 t^1/2^ (R^2^ = 0.999); MT-800 °C/GCE: Q (10^−6^C) = 0.969 + 91.84 t^1/2^ (R^2^ = 0.999).

With the Anson equation, the effective areas for the bare GCE, A/GCE, R/GCE, AR/GCE, M/GCE, MT-200 °C/GCE, MT-400 °C/GCE, MT-600 °C/GCE, and MT-800 °C/GCE were calculated as 0.2284, 0.07494, 0.03773, 0.1568, 4.6530, 2.5213, 5.9564, and 5.5964 cm^2^, respectively. The MT-600 °C/GCE displayed a relatively larger specific surface area, which was beneficial for providing more active sites for phosphoprotein detection.

The effect of different MT-600 °C composite volumes on the response current was demonstrated in Figure S16a. The oxidation peak current increased gradually with the increase volume from 1.0 to 8.0 μL, which was mainly because the more active site was produced with the increased of the amount of MT-600 °C composite. However, the current signal decreased by the further increase of MT-600 °C composite from 8.0 to 10.0 μL. This result might be ascribed to the fact that the adsorption saturation of MT-600 °C hindered the ion/electron transportation. Thus, 8.0 μL was selected as the optimal composite volume. 

To investigate the adsorption degree of 1 mg/mL β-Casein on the electrode surface, the influence of the accumulation time on the oxidation current was studied via the DPV method. As shown in Figure S16b, the peak current gradually decreased with the increase of accumulation time before 180s. However, when the accumulation time was further increased, the change in the peak oxidation current value reached a plateau, which indicated that the adsorption of β-Casein on the electrode surface reached saturation [67]. Therefore, 180 s was chosen as the most appropriate accumulation time.

The DPV method was employed to evaluate the influence of pH value, with a range from 5.0 to 9.0 of the supporting electrolytes in the electrochemical detection process. As shown in Figure S16c, the oxidation peak potentials shifted with the increased of the pH value of the detection solution, which showed that the hydrogen ion participated in electrochemical reactions. The maximum peak current was obtained at 7.0, which was selected as the optimal pH value in the follow-up experiment. 

The electrochemical kinetics reaction of β-Casein was examined on MT-600 °C/GCE via CV with different scanning rates in artificial sweat (pH = 7.0) containing 1 mg/mL β-Casein. As shown in Figure 6c,d, when the scan rate increased from 10 to 100 mV/s, the peak current of oxidation (I_pa_) and reduction (I_pc_) peak currents increased with increasing scan rate. In addition, a good linear relationship was found between the redox peak currents and square root of scan rates. The linear equations were expressed as I_pa_ = 0.6056X + 1.45565 (R^2^ = 0.99813) and I_pc_ = −0.72126X − 1.96875 (R^2^ = 0.99928), respectively. The fitting result indicated that the redox reaction of β-Casein at MT-600 °C/GCE was an adsorption-controlled process [68]. 

To evaluate the analytical performance of the sensor, the CV technique was applied to perform the different concentrations of β-Casein in artificial sweat (pH = 7.0) under optimized conditions. As shown in Figure 6e,f, the oxidation and reduction peak currents changed regularly, in accordance with the increases in the concentration of β-Casein. The concentration increased from 0.1 to 1.0 mg/mL, and the oxidation and reduction peak currents changed from −9.264 to −15.64 μA and 41.18 to 8.277 μA, respectively. The corresponding linearization equations were I_pa_ = 35.2363X + 1.70293 (R^2^ = 0.95681) and I_pc_ = −6.60012X − 8.94613 (R^2^ =0.91691). This could be attributed to the redox reaction of β-Casein in the electrolyte solution to facilitate charge transfer. The detection limit (LOD) of β-Casein was calculated to be 1.52 μM (S/N = 3), which was calculated using Equation (2), as follows [69]:

(2) $$\delta_{\mathrm{LOD}}=\frac{3\mathrm{SD}}{m}$$

where SD and m were the standard deviation for ten parallel replicates determining the blank signal and slope of the calibration curve, respectively. The analytical sensing performance of the MT-600 °C/GCE was further, compared to the previously reported β-Casein in the sweat sensor and other sensor fields. As shown in Table 1, the sensor constructed in this work showed a lower LOD. The excellent performance mainly resulted from the synergistic effects of the MXene@TiO_2_ composite and specific adsorption of the TiO_2_. 

### 3.3. Selectivity, Repeatability, Reproducibility, and Stability

#### 3.3.1. Stability

**Figure 7:**
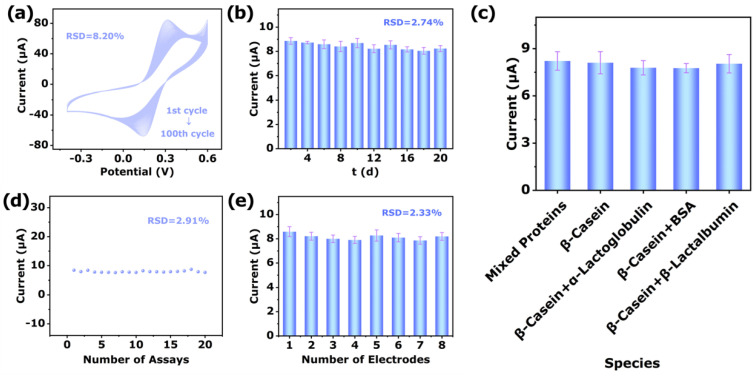
(**a**) Repeat the cycle 100 times of MT-600 °C/GCE at 100 mV/s in in 5.0 mM [Fe(CN)_6_]^3−/4−^ containing 0.1 M KCl. (**b**) CV responses current histogram of MT-600 °C/GCE in artificial sweat (pH = 7.0) containing1 mg/mL β-Casein in 20 days. (**c**) Selectivity studies of CV responses histogram of MT-600 °C/GCE. (**d**) Repetitive CV responses current histogram of MT-600 °C/GCE with one electrode for 20 times. (**e**) CV responses of MT-600 °C/GCE for eight parallel electrodes.

The cycle stability of MT-600 °C/GCE was assessed 100 times by successive CV measurement in 5.0 mM [Fe(CN)_6_]^3−/4−^ containing 0.1 M KCl (Figure 7a). The results showed that the change of the measured current relative standard deviation (RSD) value was only 8.20%, which indicated a good cycle stability.

In addition, the storage stability of MT-600 °C/GCE was evaluated by storing at room temperature in the air for 20 days, toward 1 mg/mL β-Casein, and the current value was measured every 2 days (Figure 7b). After 20 days, the sensor current RSD value was only 2.74%. The results demonstrated that the MT-600 °C/GCE had significant long-term stability.

#### 3.3.2. Selectivity

The selectivity was an indispensable index for evaluating the sensor. Thus, the selectivity of sensor toward phosphoprotein β-Casein detection was evaluated in the presence of various common non-phosphoprotein (bovine serum albumin, α-lactalbumin, and β-lactoglobulin). As shown in Figure 7c, the electrochemical responses of β-Casein and β-Casein complexed with different types of non-phosphorylated proteins were tested with a concentration of 10 mg/L as the standard. It could be clearly seen that the current intensities of β-Casein compounding other different non-phosphorylated proteins and blending all the proteins have little difference. This phenomenon indicated that the specific sensing response of MT-600 °C to β-Casein was not greatly disturbed by non-phosphoproteins. The results demonstrate that MT-600 °C/GCE has acceptable selectivity.

#### 3.3.3. Repeatability

The repeatability was studied by using one MT-600 °C/GCE under the same conditions, which were employed to measure the responding current of 1 mg/mL β-Casein 20 times successively (Figure 7d). The RSD was 2.91%, which displayed the approvable repeatability. 

#### 3.3.4. Reproducibility

The reproducibility was evaluated by using eight parallel individual MT-600 °C/GCEs under the same conditions, which were employed to detect the responding current of 1 mg/mL β-Casein (Figure 7e), respectively. The RSD of the current response was 2.33%, which showed the sensor owns satisfactory reproducibility. 

### 3.4. Sensing Mechanism Analysis

**Figure 8:**
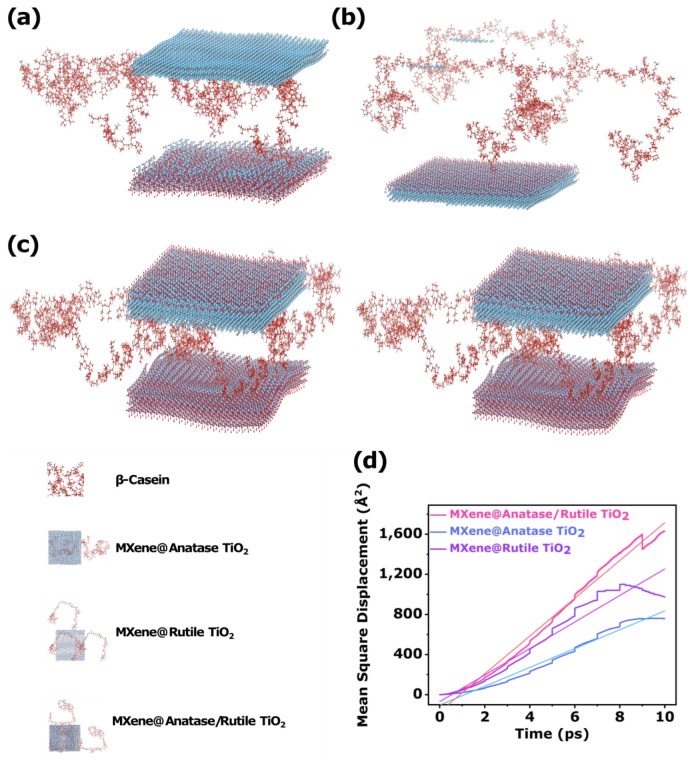
Model diagram of DFT synergistic effect of (**a**) MXene@anatase TiO_2_, (**b**)Mxene@rutile TiO_2_, and (**c**) MXene@anatase/rutile TiO_2_ heterojunction on β-Casein. (**d**) Mean square displacement during molecular dynamics simulation of MXene@anatase TiO_2_, MXene@rutile TiO_2_, and MXene@anatase/rutile TiO_2_ diffusion in β-Casein atomic structure.

Through the above studies regarding the structure and sensing properties of MXene@anatase/rutile TiO_2_, the interaction mechanism between MXene@anatase/rutile TiO_2_ ternary heterojunction and β-Casein was further elucidated. The bond length and adsorption energy changes during the adsorption process were studied by first-principles calculation. The blue, red, light blue, brown, yellow, red, and light red spheres in the ground-state molecular geometries of all computational models represented the Ti, O, N, C, S, O, and H atoms, respectively. Figure 8a–c represent the adsorption structure model diagrams for the interactions of MXene@anatase TiO_2_, MXene@rutile TiO_2_, and MXene@anatase/rutile TiO_2_ with β-Casein, respectively. 

The interactions of MXene@anatase TiO_2_, MXene@rutile TiO_2_ binary heterojunction, and MXene@anatase/rutile TiO_2_ ternary heterojunction material with β-Casein are shown in Figure 8a–c. By comparing Tables S6–S9, the difference in bond length changes after the adsorption of β-Casein between MXene@anatase TiO_2_, MXene@rutile TiO_2_ binary heterojunctions, and MXene@anatase/rutile TiO_2_ ternary heterojunctions can be seen. It can also be seen that the bond length changes of the three heterojunction systems for β-Casein can be ordered as MXene@anatase/rutile TiO_2_ > MXene@anatase TiO_2_ > MXene@rutile TiO_2_. This indicates that the MXene@anatase/rutile TiO_2_ ternary heterojunction was the best for β-Casein enrichment detection. This could be attributed to the existence of TiO_2_ n-n-type heterojunction nanoparticles with an anatase/rutile composite phase on the surface of MXene, besides abundant multi-affinity sites, such as many Ti-O. This could further improve the affinity of the MXene surface for β-Casein. The Ti-O bonds could selectively enrich and detect phosphoproteins by strongly chelating the phosphate groups in the β-Casein structure. At the same time, the carriers had better spatial separation at the phase interface in MXene and anatase/rutile TiO_2_ heterojunction materials, and the ternary synergy was beneficial for fast electron transfer. Therefore, with the synergistic effect of multi-affinity sites in the MXene@anatase/rutile TiO_2_ ternary heterojunction, the superiority of β-Casein sensing and detection was further verified. Moreover, the adsorption energies of the three heterojunction sensing systems and β-Casein were calculated, as shown in Table S10. The adsorption energies of the three heterojunction sensing systems were all negative and could be ranked as MXene@anatase/rutile TiO_2_ > MXene@anatase TiO_2_ > MXene@rutile TiO_2_. This indicated that the adsorption process of MXene@anatase/rutile TiO_2_ to β-Casein was an exothermic and stable process.

The molecular dynamics simulation of MXene@anatase TiO_2_, and MXene@rutile TiO_2_ binary heterojunction, and MXene@anatase/rutile TiO_2_ ternary heterojunction system models could be described by the mean square displacement (MSD). The MSD curves with higher slopes had higher molecular mobility, which was favorable for the efficient movement of β-Casein molecules in the heterojunction structure. The MSD could be defined as the statistical square of the offset of all particles in the system, relative to their initial positions at a certain moment, which can be expressed as Equation (3) [78]:

(3) $$\mathrm{MSD}=\langle {\left|r\left(t\right)-r\left(0\right)\right|}^{2}\rangle$$

where “< >” represents the average of all atoms and initial configurations in the crystal structure, and MSD is the mean square displacement; r(t) and r(0) represent the molecular coordinates at the moment and initial molecular coordinates, respectively.

The MSD-t (mean square displacement-time) curves of different molecular structures in three heterojunction sensing systems and β-Casein atomic structure are shown in Figure 8d. It could be clearly seen that, in the simulated time system, the MSD curves of the three heterojunction materials approximately exhibited a linear relationship, and the MSD value increased linearly with the increase of the simulation time. According to the trend of the curve in the Figure 8d, it can be seen that the MSD slope of the MXene@anatase/rutile TiO_2_ ternary heterojunction was significantly larger than that of the MXene@anatase TiO_2_ and MXene@rutile TiO_2_ binary heterojunctions. This indicates that, under the same test conditions, with the layer-by-layer in situ construction of the heterojunction, the intensified movement of the β-Casein molecules in MXene@anatase/rutile TiO_2_ could be promoted, which increased the degree of spreading of the β-Casein molecule in the ternary heterojunction structure [79]. Therefore, the β-Casein molecule had stronger diffusivity in the MXene@anatase/rutile TiO_2_ ternary heterojunction than the other two binary heterojunctions. This indicates that the anatase/rutile TiO_2_ binary heterojunction generated in situ on the MXene surface in the ternary heterojunction could improve the stretchability of the molecular chain of β-Casein in the MXene@anatase/rutile TiO_2_. In addition, the mobility of the molecular chain was improved, thereby improving the sensing performance of the ternary system for β-Casein.

In addition, to further study the intrinsic reasons for the detection of β-Casein by three heterojunction materials, the diffusion and movement abilities of β-Casein molecule in the MXene@anatase TiO_2_, MXene@rutile TiO_2_, and MXene@anatase/rutile TiO_2_ structure were further quantitatively studied. Specifically, the corresponding slope obtained by linear fitting of the MSD curve was substituted into the law of molecular diffusion coefficient, and the diffusion coefficient (D) was further calculated by Equation (4) [80]:

(4) $$D=\underset{x\to \infty }{\lim}\frac{\langle \left|r\left(t\right)-r\left(0\right)\right|\rangle }{6t}=\frac{s\left(t\right)}{6t}=\frac{m}{6}$$

where m represents the slope of the MSD. The diffusion coefficient can be approximated as 1/6 of the MSD slope. According to this method, the diffusion coefficients of the three heterojunction sensing materials were shown in Table S11, which clearly reflected the mobility of the β-Casein molecules in the three heterojunction structures. By sorting the diffusion coefficients of the three heterojunction systems, the result was MXene@anatase/rutile TiO_2_ > MXene@anatase TiO_2_ > MXene@rutile TiO_2_. Among them, the diffusion coefficient of MXene@anatase/rutile TiO_2_ ternary heterojunction system was the largest, which indicates that β-Casein protein molecules in MXene@anatase/rutile TiO_2_ had stronger diffusivity and could better respond to β-Casein.

It can be seen that the TiO_2_ molecules of the anatase/rutile composite phases on the 2D structure of MXene could effectively promote the further migration ability of the β-Casein protein molecules in the ternary heterojunction and increase the diffusion coefficient. In addition, the β-Casein protein molecules entered the MXene@anatase/rutile TiO_2_ ternary heterojunction structure at the fastest speed through molecular diffusion and were least affected by the hindering of the atomic chain of the ternary heterojunction system. These were the intrinsic reasons for promoting the β-Casein sensing performance of the MXene@anatase/rutile TiO_2_ ternary heterojunction [81]. 

### 3.5. Practical Application Sensor Evaluation

**Figure 9:**
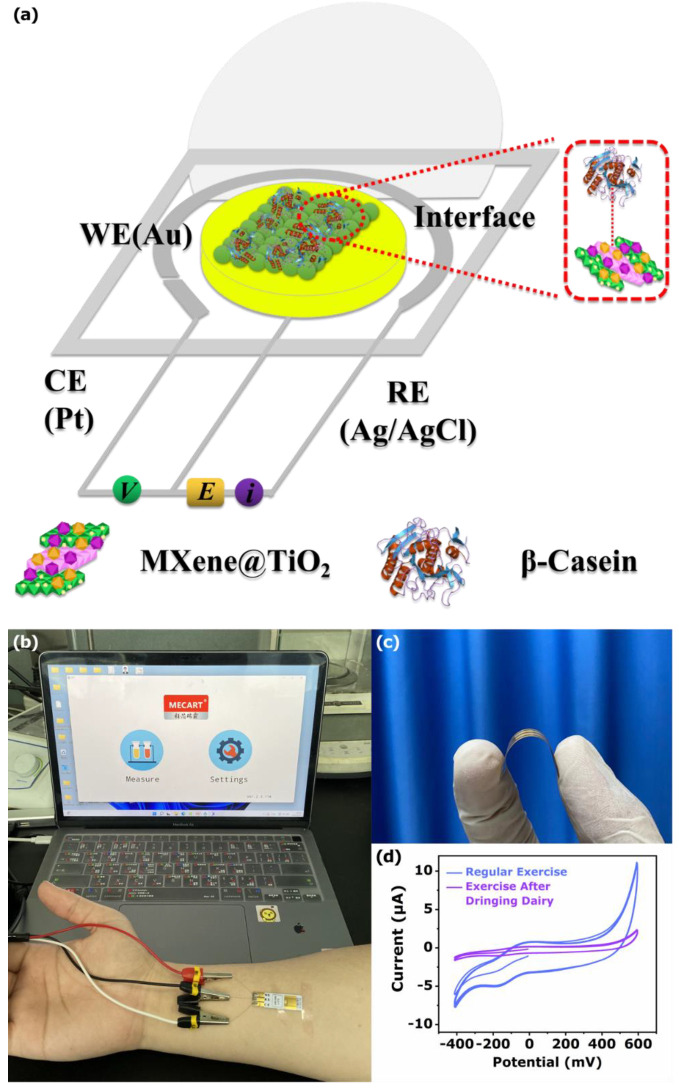
(**a**) Schematic diagram of wearable sweat sensor array. (**b**) Optical photo for real-time, on-body sweat monitoring. (**c**) PI flexible sensing platform. (**d**) Data-monitoring chart under different exercise conditions.

To verify the accuracy of this sensing method, the monitoring of sweat phosphoproteins in practical applications was realized by a flexible substrate. Precisely, the sensing chip was a flexible substrate chip based on Au (working electrode), Ag/AgCl (reference electrode), and Pt (counter electrode). The construction of the device was mainly through the spin coating method to construct the MT-600 °C sensing array on the surface of the sensing chip (Figure 9a). The PI chip was attached to the volunteer’s curved arm, without any separation (Figure 9b). Additionally, Figure 9c demonstrates the bendability of the flexible substrate. An application of the classic CV response of the sensor was acquired when a person cycled in a closed room for 15 min while drinking 100 mL of dairy products. In addition, the contrastive CV response was obtained from another person only cycling for 15 min. The CV response of the latter was significantly greater than that of the former (Figure 9d). This phenomenon could be attributed to the macromolecular phosphoprotein metabolized from the sweat adsorbed on the surface of MT-600 °C. These results indicate that the constructed phosphoprotein electrochemical sensor was potentially applicable for phosphoprotein in the sweat in the real sample. These results indicate that the MT-600 °C sensing material was potentially applicable for phosphoprotein analysis in the sweat sample.

## 4. Conclusions

In summary, a sensitive and selective electrochemical sensing platform was fabricated for phosphoprotein detection in sweat via in situ calcination growth anatase/rutile TiO_2_ on the surface of MXene, in order to construct an intercalation structure MXene@anatase/rutile TiO_2_. The intercalation structure MXene@anatase/rutile TiO_2_ effectively provided abundant adsorption activity toward phosphoprotein, due to the huge surface area, high conductivity, excellent mass transfer ability with accordion-shaped MXene, and great adsorption capacity from anatase/rutile TiO_2_, and could efficiently capture phosphoprotein to greatly amplify the detection signal. Consequently, the as-prepared sensor demonstrated excellent performance, with a wide linear relationship, ranging from 0.01 to 1 mg/mL, with a low LOD of 1.52 μM. Additionally, the sensing platform showed satisfactory selectivity, repeatability, reproducibility, and stability. Subsequently, the response mechanism of MXene@anatase/rutile TiO_2_ ternary heterojunction for phosphoprotein detection was investigated by first-principles calculations, and the results showed that the specific enrichment of ternary heterojunctions for the detection of phosphoproteins was due to the phase–interface interaction of the layer-by-layer heterojunctions and existence of abundant Ti-O group affinity sites. Then, the diffusion motion of phosphoprotein molecules in the MXene@anatase/rutile TiO_2_ ternary heterojunction structure was simulated by molecular dynamics, which further proved the good spreading degree and mobility of the phosphoprotein molecules in the heterojunction structure. Additionally, the constructed sensor was successfully used for the detection of phosphoproteins in sweat during exercise, which indicated that the developed strategy could be readily extended for phosphoprotein detection in sweat. Therefore, future efforts can involve combining this ternary heterojunction sensing material with wearable, microfluidic, and integrated circuit printing technologies for the fabrication of portable, wearable sweat sensors to achieve a wide range of high-performance, modern, and timely diagnoses.

## Figures and Tables

**Table 1:** Comparison of analytical performance toward phosphoprotein detection in different fields.

Samples	Modified Materials	Liner Range	LOD	References
Sweat	g-C_3_N_4_@Fe_3_O_4_	0.01–1 mg/mL	9.7 μM	[70]
Water	Zn^II^-DPA ^a^		0.22 ppm	[71]
Electrolyte	DPA-NH_2_^b^	≥1 nM		[72]
Electrolyte	DPA-Zn^2+^ ^c^	≥1 nM		[73]
Glioblastoma cell	Silicon photonic microring resonator arrays	3.55−log	0.6 pM	[74]
Cancer cell	Zr-FeTCPP ^d^-MOF	0.1–40 nM/40–150 nM		[75]
Food	NH_2_-TiO_2_/UCNPs ^e^-rGO	0–1 mg/mL	9.2 × 10^−5^ mg/mL	[76]
Food	NH_2_-TiO_2_/MUA ^f^/AuE ^g^-QCM ^h^	1.0 × 10^−3^–1.0 mg/mL	0.09 mM	[77]
Sweat	MXene@TiO_2_	0.01–1 mg/mL	1.52 μM	This work

^a^: Zinc (II)-dipicolylamine; ^b^: 4-[bis(2-pyridylmethyl) aminomethyl] aniline; ^c^: dipicolylamine–zinc chelates 4; ^d^: Fe (III) meso-tetra (4-carboxyphenyl) porphine 6; ^e^: upconversion nanomaterials; ^f^: 11-mercaptoundecanoic acid; ^g^: Au electrode; ^h^: quartz crystal microbalance.

## Data Availability

Not applicable.

## Supplementary Materials

The following supporting information can be downloaded at: https://www.mdpi.com/article/10.3390/bios12100865/s1. References [82,83,84,85,86,87,88,89,90,91,92,93,94,95,96,97,98,99,100,101] are cited in the supplementary materials.
