# Supplementary material for: In Situ Growth Intercalation Structure MXene@Anatase/Rutile TiO2 Ternary Heterojunction with Excellent Phosphoprotein Detection in Sweat
Source: Biosensors (Basel). 2022 Oct 12;12(10):865. doi: 10.3390/bios12100865 (PMC9599406; doi:10.3390/bios12100865)
Supplement: Supplementary file 1 [file biosensors-12-00865-s001.zip › biosensors-1937832-supplementary.pdf]

biosensors

Supporting Information

In Situ Growth Intercalation Structure MXene@Anatase/Rutile TiO_2 Ternary Heterojunction with Excellent Phosphoprotein Detection in Sweat

Yuting Qiao [1], Xianrong Liu [1], Zhi Jia [1], Peng Zhang [1], Li Gao [1,*], Bingxin Liu [1,*], Lijuan Qiao [2,*] and Lei Zhang [3]

[1] School of Mechanical Engineering, Qinghai University, Xining 810016, China
[2] Research Center of Basic Medical Science, Medical College, Qinghai University, Xining 810016, China
[3] Department of Mechanical Engineering, University of Alaska Fairbanks, Fairbanks 755905, AK, USA
* Correspondence: 2007990030@qhu.edu.cn (L.G.); liubx408@nenu.edu.cn (B.L.); 2014980007@qhu.edu.cn (L.Q)

Materials and Methods

Synthesis of Different Crystalline Phase TiO_2

A total of 2 g of anatase TiO_2 powder was placed into crucibles and muffle furnaces, which were heated to 600, 650, 700, 750, and 800 °C at a heating rate of 5 °C/min, under an air atmosphere, in order to obtain TiO_2 powders with different crystalline phase, respectively.

Computation Methods

DFT calculation was based on density functional theory (DFT) of the first principle molecular dynamics calculation method, calculation software VASP [1,2]. The exchange correlation potential was approximated by the generalized gradient of the Perdew-Wang91 form [3]. The projection augmented plane wave (PAW) method was selected to describe the interaction between electrons and ions [4]. The cut off energy of plane wave was 400 eV. Spin was considered in all calculations. The energy convergence criterion between the two electron steps was 10^{-4}eV. In the surface calculation, p(5 × 4) supercells were used to sample the Brillouin zone, with a K-grid centered on a 1 × 1 × 1 Γ point. Atomic coordinates are optimized until the maximum force applied to each atom was no greater than 0.01 eVÅ^{-1}. To avoid interaction between adjacent periodic structures, a vacuum layer of more than 45 Å was created in the c direction. The convergence criterion for protein adsorption was 0.05 eVÅ^{-1}. The NVT ensemble was used to investigate the first-principles molecular dynamics calculation in this paper. The calculation time step was 1 fs, and the total simulation time was 100 ps.

In addition, the first-principles molecular dynamics (AIMD) simulation was completed based on VASP software, and the mean square displacement (MSD) was calculated using Vaspkit software.

Reference

[1] Kresse, G.; Furthmüller, J. Efficiency of ab-initio total energy calculations for metals and semiconductors using a plane-wave basis set-ScienceDirect. *Comput. Mater. Sci.* **1996**, *6*, 15–50.

[2] Kresse, G.; Hafner, J. Ab initio molecular dynamics for liquid metals. *Phys. Rev. B* **1993**, *47*, 13115–13118.

[3] Perdew, P.; Wang, Y. Accurate and simple analytic representation of the electron-gas correlation energy. *Phys. Review. B* **1992**, *45*, 13244–13249.

[4] Blöchl, E. Projector Augmented-Wave Method. *Phys. Rev. B* **1994**, *50*, 17953–17979.

Results and Discussions

Characterization of The Sensing Materials

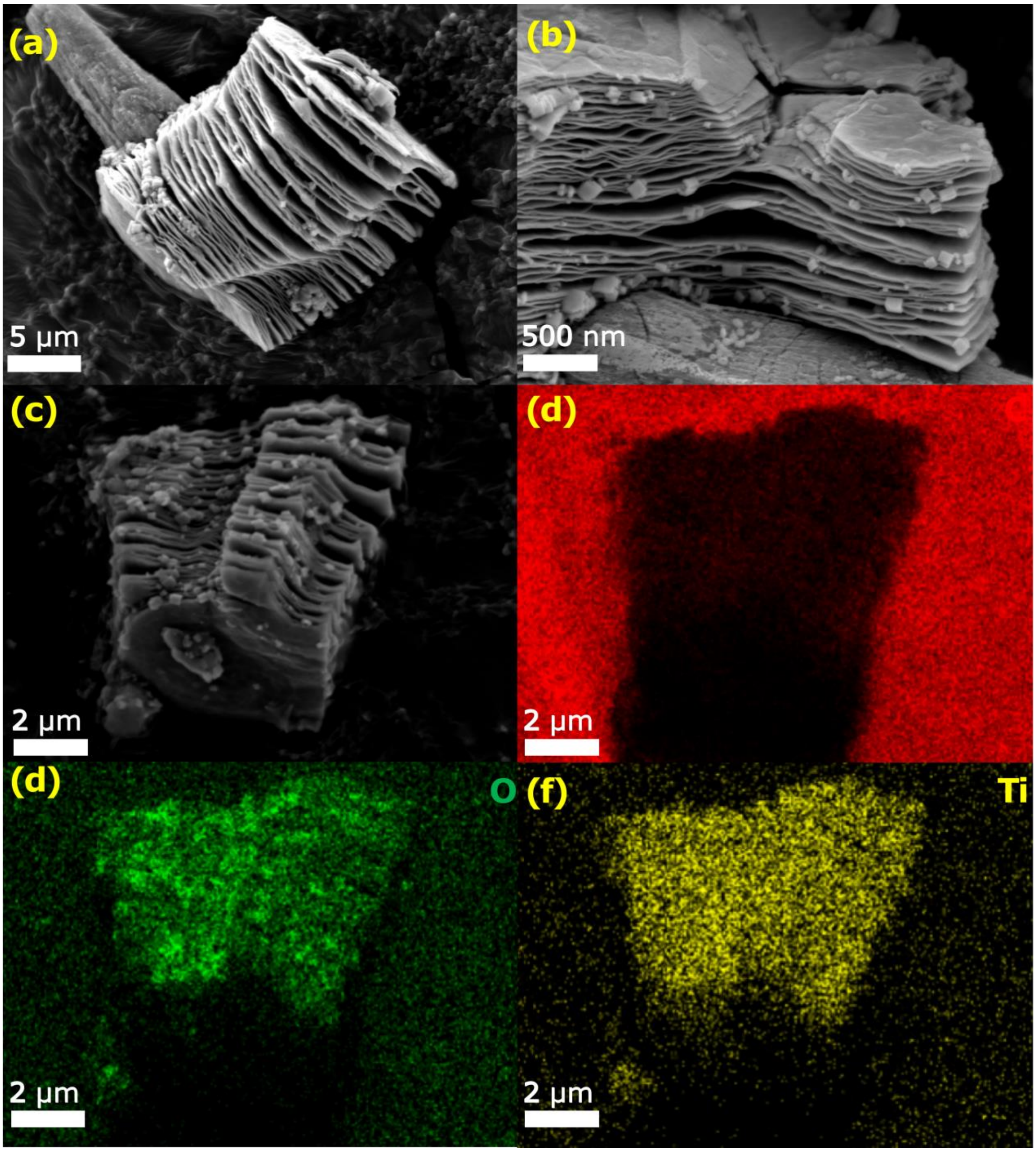

Figure S1. SEM images of (**a**)–(**c**) MT-200 °C under different angles and scan magnification and mappings of (**d**) C, (**e**) O, and (**f**) Ti elements.

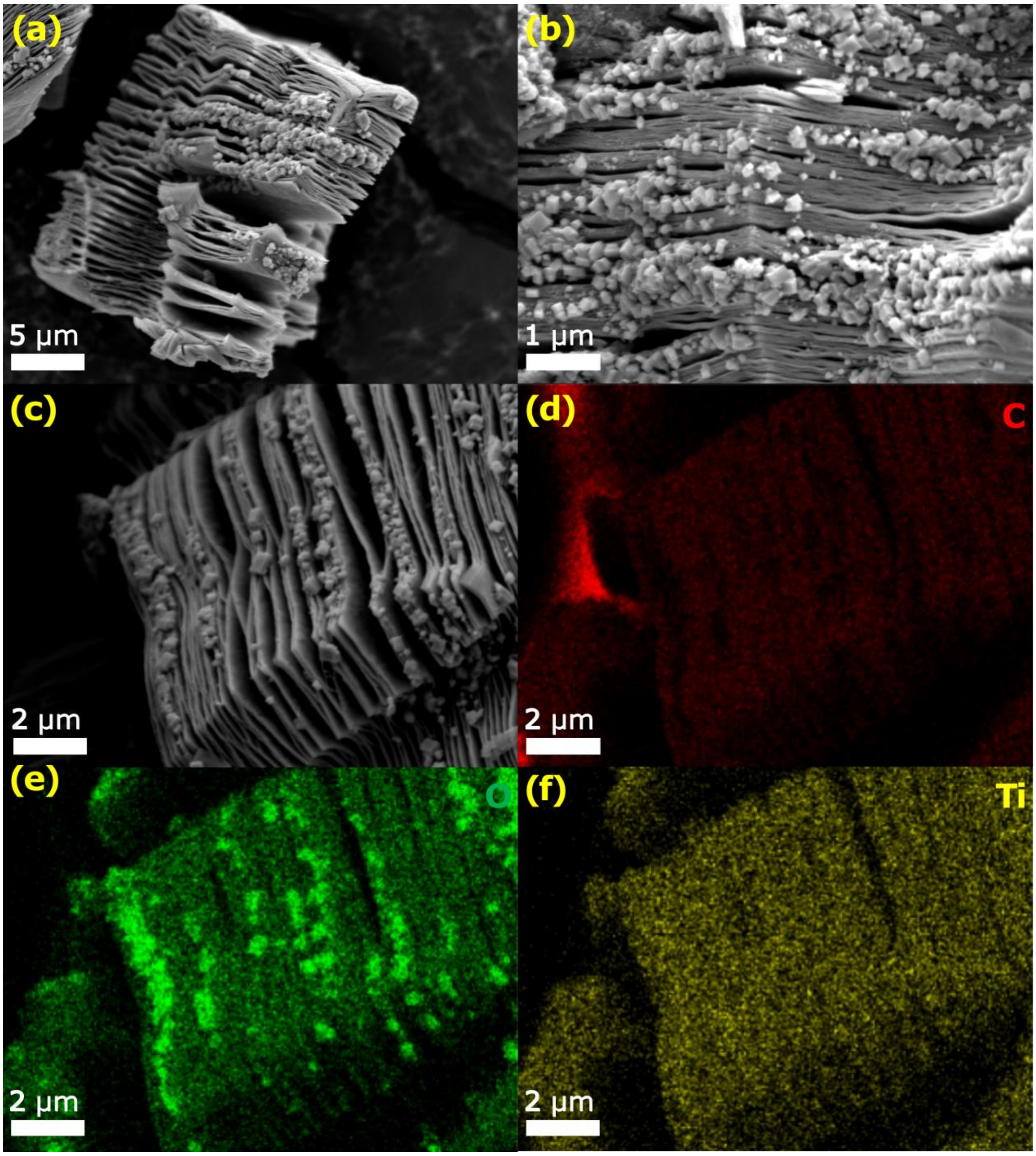

Figure S2. SEM images of (**a**)–(**c**) MT-400 °C under different angles and mappings of (**d**) C, (**e**) O, and (**f**) Ti elements.

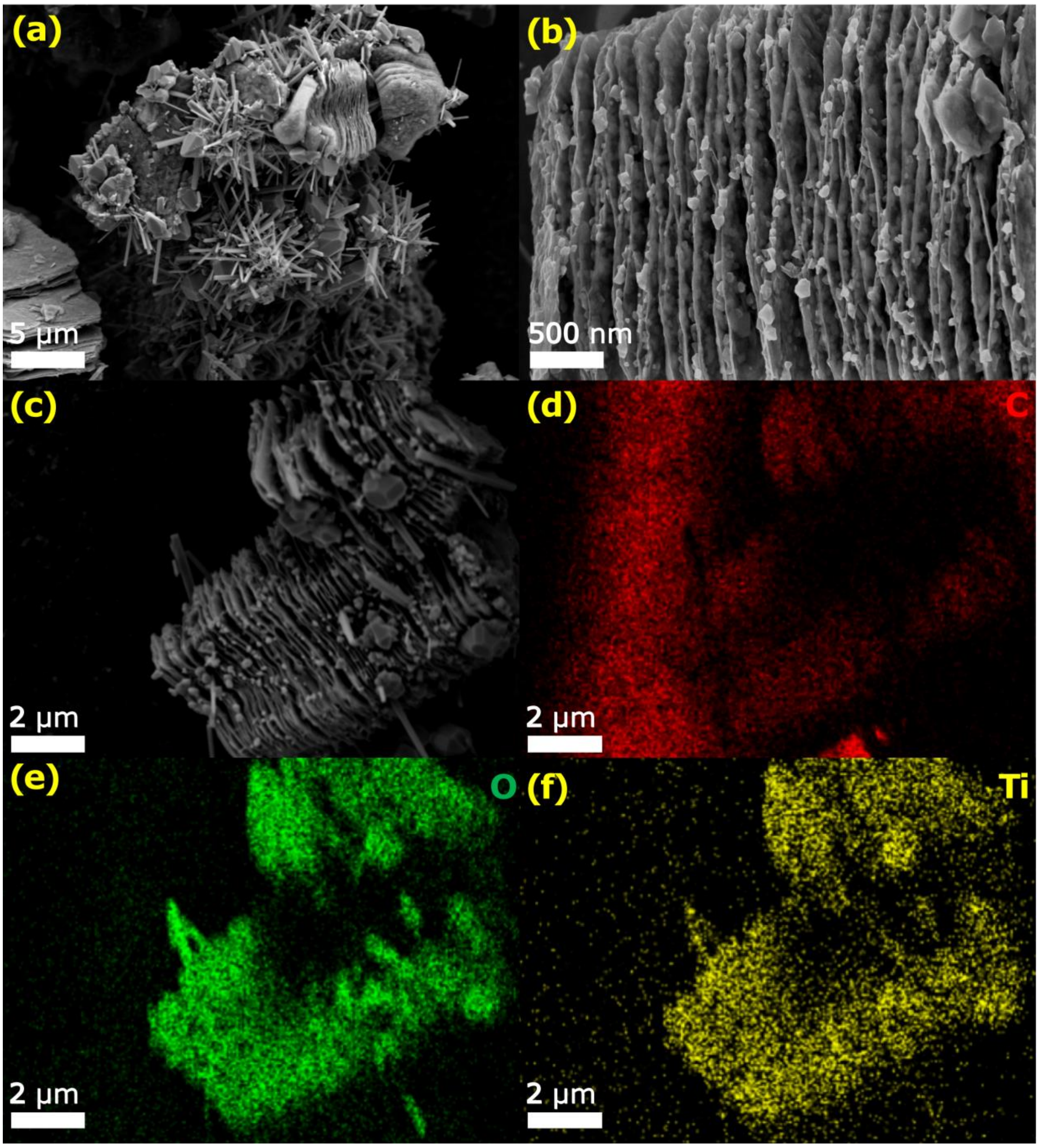

Figure S3. SEM images of (**a**)–(**c**) MT-800 °C under different angles and scan magnification and mappings of (**d**) C, (**e**) O, and (**f**) Ti elements.

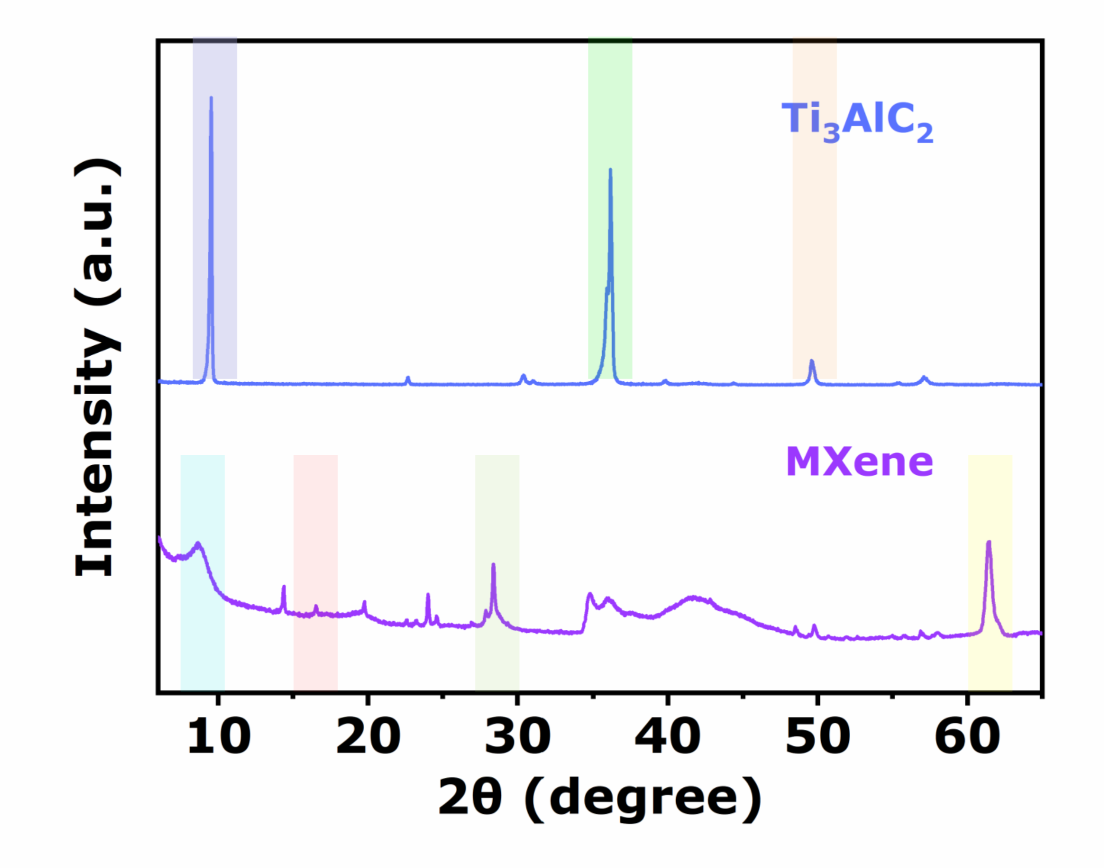

Figure S4. XRD patterns of Ti_3AlC_2 and MXene.

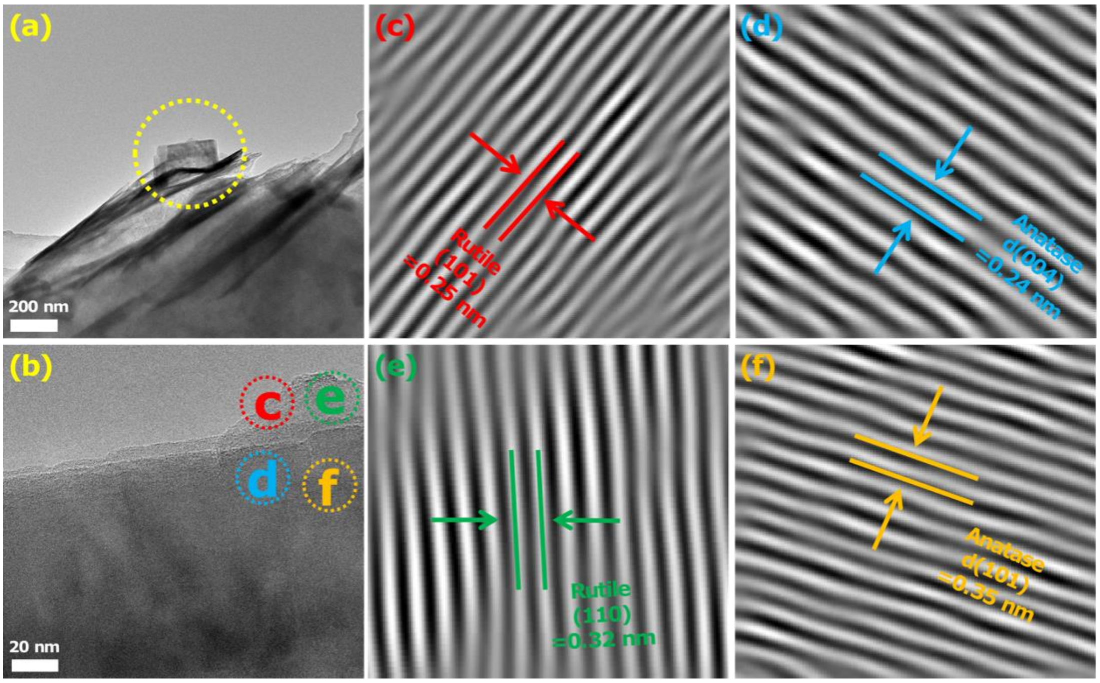

Figure S5. (**a**)–(**b**) HRTEM of MT-600 °C and the corresponding spacing images of (**c**)–(**f**) TiO_2 (anatase and rutile) planes with the selected area.

Figure S5 clearly shows the TEM image of the anatase/rutile TiO_2 structure in situ generated on the MXene layer (Figure S5a). Further HRTEM revealed lattice fringes of TiO_2 (Figure S5b), where the lattice spacings of 0.25, 0.24, 0.32, and 0.35 nm could specifically corresponded to rutile (101), anatase (004), rutile (110), and anatase phase (101) lattice fringes (Figure S5c–f).

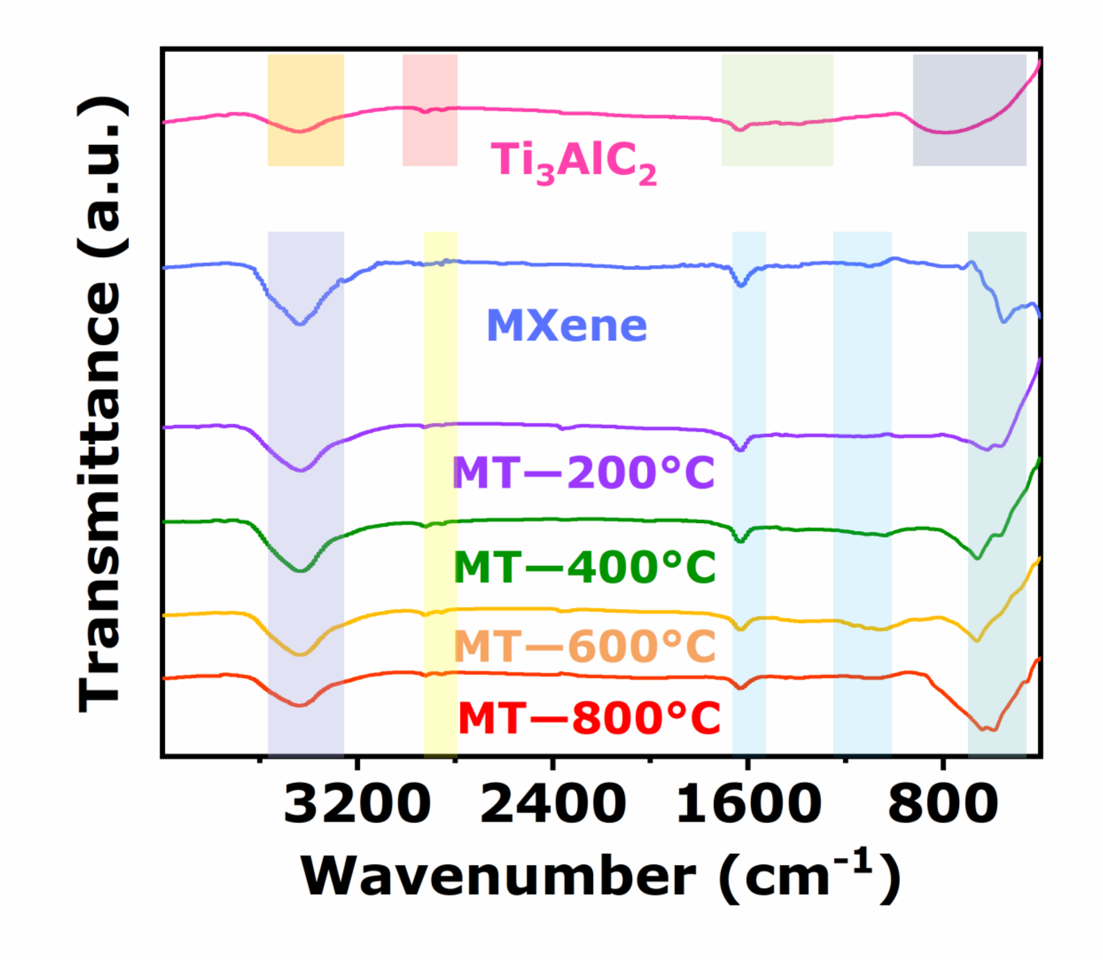

Figure S6. FT-IR patterns of Ti_3AlC_2, MXene, MT-200 °C, MT-400 °C, MT-600 °C, and MT-800 °C.

The Fourier transform infrared spectroscopy (FT-IR) spectrum was used to verify the chemical structure and surface functional groups of sensing materials, as shown in Figure S6. In the curve of Ti_3AlC_2, the characteristic peaks at 3475 and 2927 cm^{-1} were the stretching vibrational absorption peaks of -OH groups. Additionally, the characteristic peaks located at 1632 and 784 cm^{-1} were the stretching vibration absorption peaks of C=O and Ti-O [1]. In addition, three strong absorption peaks at 3420, 2927, and 1632 cm^{-1} of MXene belonged to the -OH stretching, C–H stretching, and C=O stretching vibrational peaks. Comparing Ti_3AlC_2 and MXene, it can be seen that the stretching vibration peak of -OH on the surface of MXene was sharper and more prominent. The reason was that the amount of -OH increase after HF etching, which was beneficial to the next in situ oxidation [2]. Furthermore, the broad absorption peak at 3434 cm^{-1} was attributed to the -OH adsorbed on titanium atom (Ti-OH) after calcination of MXene at different temperatures. The gradually weakening absorption peaks around 2927 and 1632 cm^{-1} corresponded to the O-H stretching vibration, which indicated that many -OH groups were terminated on the MXene surface. After the calcination oxidation, a strong absorption peak appeared near 660 cm^{-1}, which could be attributed to the Ti-O-Ti stretching vibration of TiO_2 [3].

Reference

[1] Ren, S.; Feng, R.; Cheng, S.; Wang, Q.; Zheng, Z. Synergistic Catalytic Acceleration of MXene/MWCNTs as Decorating Materials for Ultrasensitive Detection of Morphine. *Electroanal.* **2021**, *33*, 1471–1483.

[2] Zhou, L.; Wu, F.; Yu, J.; Deng, Q.; Zhang, F.; Wang, G. Titanium carbide ($Ti_3C_2T_x$) MXene: A novel precursor to amphiphilic carbide-derived graphene quantum dots for fluorescent ink, light-emitting composite and bioimaging. *Carbon* **2017**, *118*, 50–57.

[3] Peng, C.; Wang, H.; Yu, H.; Peng, F. (111) TiO_{2-x}/Ti_3C_2: Synergy of active facets, interfacial charge transfer and Ti^{3+} doping for enhance photocatalytic activity. *Mater. Res. Bull.* **2017**, *89*, 16–25.

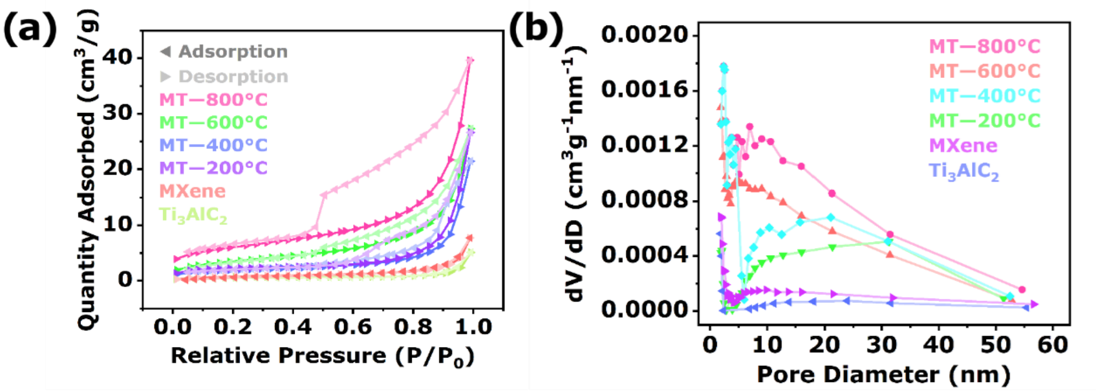

Figure S7. (**a**) N_2 adsorption-desorption isotherms and (**b**) pore distribution curve of Ti_3AlC_2, MXene, MT-200 °C, MT-400 °C, MT-600 °C, and MT-800 °C.

To study the effects of calcination temperatures on sensing materials, the N_2 adsorption-desorption isotherms are depicted in Figure S7a and S7b, and the data are displayed in Table S5. The N_2 adsorption-desorption isotherm of MXene was represented by the type IV isotherm, and there was an obvious H3-type hysteresis loop [1]. Similarly, the Ti_3AlC_2 showed an obvious H3-type hysteresis loop and had the existence of microporous structure. The specific surface, pore volume, and pore diameter of Ti_3AlC_2 and MXene were 1.0892 m^2/g, 0.007898 cm^3/g, 17.4197 nm, 2.3621 m^2/g, 0.011941 cm^3/g, and 23.9462 nm. It could be clearly seen that the physicochemical parameters of MXene were significantly better than Ti_3AlC_2. The reason was that the Al atoms were etched to form two-dimensional MXene with interlayer diffusion during the etching process [2]. In addition, the specific surface area of MXene was relatively lower than that of MXene@TiO_2 at different temperatures. The main reason was that the structure of the MXene layer formed during the Ti_3AlC_2 exfoliation process was unstable, which led to a certain degree of stacking to occur, and part of the structure bent. Moreover, many functional groups were adsorbed on the surface of MXene, which would also have a certain effect on the interlayer spacing. This result was consistent with the results of XRD [3]. When MXene was treated at different temperatures, type IV isotherm with H3 hysteresis loops were observed on all MXene@TiO_2 under different temperatures, due to the presence of mesopores and micropores [4]. Additionally, the specific surface areas of MXene@TiO_2 were 7.5112 m^2/g (MT-200 °C), 8.2860 m^2/g (MT-400 °C), 12.7380 m^2/g (MT-600 °C), and 21.2380 m^2/g (MT-800 °C). Herein, the increase in specific surface area could be attributed to the higher proportion of TiO_2 with different sizes and different crystal types grown in situ on the surface and interior of MXene with increasing calcination temperature [5]. The larger specific surface area of the composites could provide more transport channels capable of adsorbing protein molecules, which would be beneficial for improving the sensing performance. Additionally, the pore volumes were 0.042259 (MT-200 °C), 0.033183 (MT-400 °C), 0.040121 (MT-600 °C), and 0.061318 (MT-800 °C) cm^3/g, respectively. In addition, the pore diameter sizes were 26.1093 (MT-200 °C), 24.8589 (MT-400 °C), 14.3820 (MT-600 °C), and 11.5487

(MT-800 °C) nm. In addition, the changes in pore volume and diameter sizes indicated that the initial calcination and carbonization under the MXene material framework could gradually be formed a microporous structure, and the newly formed micropores were destroyed as the temperature increased to form a mesoscopic structure. Additionally, the changes in total pore volume were irregular, mainly caused by changes in the micropore/mesopore ratio [6].

Reference

[1] Liu, M.; Li, J.; Bian, R.; Wang, X.; Ji, Y.; Zhang, X.; Tian, J.; Shi, F.; Cui, H. ZnO@Ti_3C_2 MXene interfacial Schottky junction for boosting spatial charge separation in photocatalytic degradation. *J. Alloy. Compd.* **2022**, *905*, 164025.

[2] Dong, X.; Wang, Y.; Jia, M.; Niu, Z.; Cai, J.; Yu, X.; Ke, X.; Yao, J.; Zhang, X. Sustainable and scalable in-situ synthesis of hydrochar-wrapped Ti_3AlC_2-derived nanofibers as adsorbents to remove heavy metals. *Bioresour. Technol.* **2019**, *282*, 222–227.

[3] Wang, K.; Zhou, Y.; Xu, W.; Huang, D.; Wang, Z.; Hong, M. Fabrication and thermal stability of two-dimensional carbide Ti_3C_2 nanosheets. *Ceram. Int.* **2016**, *42*, 8419–8424.

[4] Li, A.; Wang, Z.; Yin, H.; Wang, S.; Yan, P.; Huang, B.; Wang, X.; Li, R.; Zong, X.; Han, H.; Li, C. Understanding the anatase-rutile phase junction in charge separation and transfer in a TiO_2 electrode for photoelectrochemical water splitting. *Chem. Sci.* **2016**, *7*, 6076–6082.

[5] Xia, X.; Peng, S. Bao, Y.; Wang, Y.; Lei, B.; Wang, Z.; Huang, Z.; Gao, Y. Control of interface between anatase TiO_2 nanoparticles and rutile TiO_2 nanorods for efficient photocatalytic H_2 generation. *J. Power Sources* **2018**, *376*, 11–17.

[6] Chen, J.; Zhang, X.; Bi, F.; Zhang, X.; Yang, Y.; Wang, Y. A facile synthesis for uniform tablet-like TiO_2/C derived from Materials of Institut Lavoisier-125(Ti) (MIL-125(Ti)) and their enhanced visible light-driven photodegradation of tetracycline. *J. Colloid Interface Sci.* **2020**, *571*, 275–284.

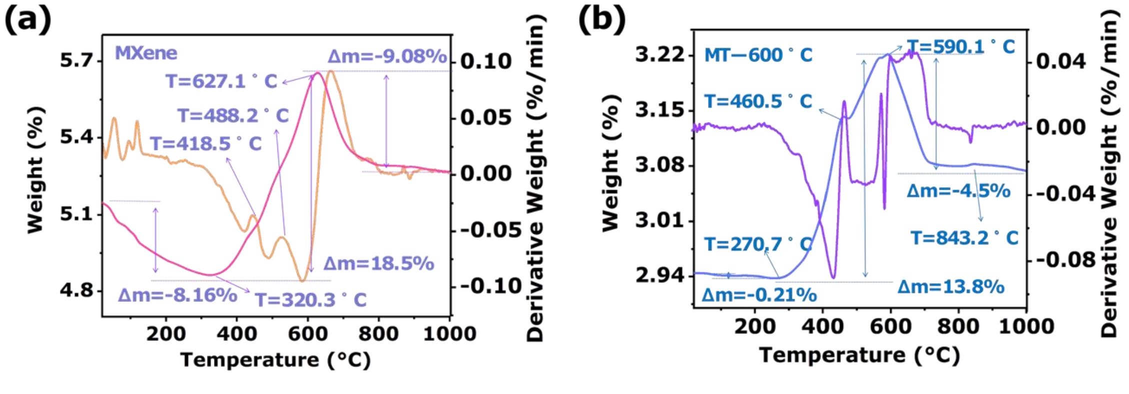

Figure S8. TG/DSC curves of (a)MXene, and (b)MT-600 °C.

The thermal stability performance of the sensing materials was studied under the air atmosphere with 10 °C/min heating rate in the range of room temperature (RT) to 1000 °C. As shown in Figures S8a and S8b, the thermogravimetric decomposition curves of both MXene and MT-600 °C could be divided into three stages. The materials weight loss in the first stage was about 8.16% and 0.21% weight loss from RT to 320.3 and 270.7 °C, respectively, which could be attributed to the loss of physical adsorbed water molecules on the surface of MXene and MT-600 °C [1]. The second stage oxidations of MXene and MT-600 °C were from 320.3 to 627.1 °C and 270.7 to 590.1 °C, with weight gains of about 18.5% and 13.8%, respectively. This stage in MXene was accompanied by a very strong exothermic process, so the MXene began to oxidize heavily, and the weight of the sample

increased dramatically. The anatase and rutile phases of TiO_2 were gradually formed during the calcination and oxidation of MXene. The research shows that rutile phase TiO_2 could be further transformed from anatase phase TiO_2 [2]. Therefore, the anatase phase TiO_2 was first gradually oxidized and formed between the surface and interior of the MXene in the second stage. However, the internal anatase phase TiO_2 blocked the diffusion of O_2 and slowed further oxidation [3]. With the slow diffusion of O_2 at high temperature, TiO_2 was oxidatively transformed from anatase phase to rutile phase. This process in MXene corresponded to two relatively weak exothermic peaks at 418.5 and 488.2 °C. Likewise, a small amount of anatase phase TiO_2 in MT-600 °C was completely converted into rutile phase TiO_2 at this stage. Additionally, the relatively strong exothermic peak at 460.5 °C corresponded to the further oxidation of anatase phase TiO_2 caused by O_2 diffusion at a high calcination temperature. The sustained weight loss in the third stage of MXene and MT-600 °C corresponded to the weight loss state of 627.1 and 590.1 °C to 1000 °C. This stage was mainly due to the conversion of C element in MXene into volatile species CO_2 and chemical absorption of -OH and -F, which promoted further weight loss of the material [4].

Reference

[1] Wang, K.; Zhou, Y.; Xu, W.; Huang, D.; Wang, Z.; Hong, M. Fabrication and thermal stability of two-dimensional carbide Ti_3C_2 nanosheets. *Ceram. Int.* **2016**, *42*, 8419–8424.

[2] Zhu, S.; Xie, S.; Liu, Z. Nature of Rutile Nuclei in Anatase-to-Rutile Phase Transition. *J. Am. Chem. Soc.* **2015**, *137*, 11532–11539.

[3] Li, Z.; Wang, L.; Sun, D.; Zhang, Y.; Liu, B.; Hu, Q.; Zhou, A. Synthesis and thermal stability of two-dimensional carbide MXene Ti_3C_2. *Mater. Sci. Eng. B* **2015**, *B191*, 33–40.

[4] Aihu, F.; Yun, Y.; Feng, J.; Yong, W.; Le, M.; Yang, Y.; Li, S. Fabrication and thermal stability of NH_4HF_2-etched Ti_3C_2 MXene. *Ceram. Int.* **2017**, *43*, 6322–6328.

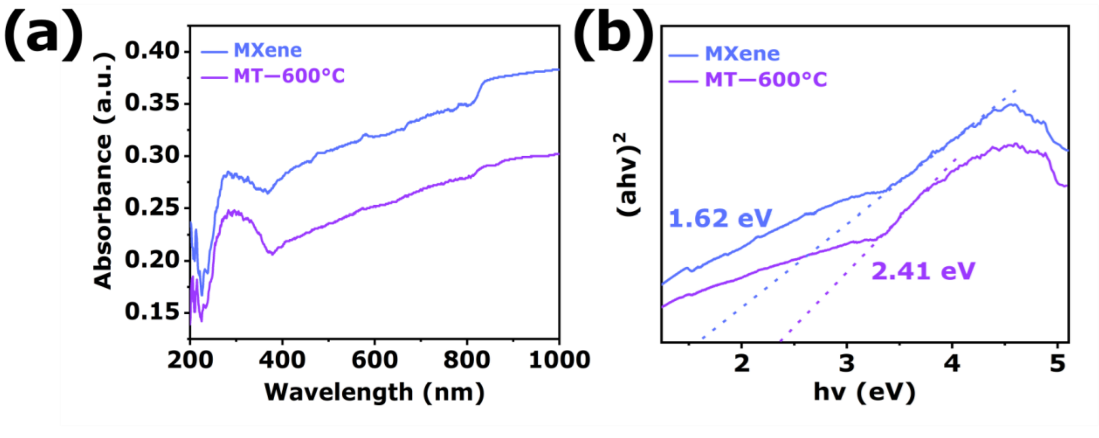

Figure S9. (**a**) UV–Vis diffuse reflectance spectra and (**b**) Tauc plot of band gaps determination of the MT-600 °C.

The UV–Vis diffuse reflectance spectra (DRS) of the as-fabricated samples were shown in Figures S9a and S9b, from 200 to 1000 nm. The absorption range of MXene and MT-600 °C included the ultraviolet and visible regions. It is worth noting that both exhibited strong UV absorption and broad characteristic peaks in the UV–vis spectra, from 750 to 850 nm. This characteristic peak was distinct and different from traditional two-dimensional materials, and this interval window was widely suitable for biomedical applications [1]. By calculating the corresponding band gap energies of MXene and MT-600 °C sensing materials, the band gap values of MXene and MT-600°C were 1.62 and 2.41 eV. Compared with the band gap value (3.27 eV) of the TiO_2 heterojunction in study, this

showed that the construction of the heterojunction obviously optimized the electronic energy band structure of the sensing material [2,3].

Reference

[1] Lin, H.; Wang, X.; Yu, L.; Chen, Y.; Shi, J. Two-Dimensional Ultrathin MXene Ceramic Nanosheets for Photothermal Conversion. *Nano Lett* **2017**, *17*, 384–391.

[2] Yu, J.; Zhang, P.; Chen, T.; Lv, Q.; Gao, L.; Liu, B.; Duan, J.; Wu, Z.; Li, J. Construction of flexible and wearable 3D TiO_2 NTs@Ti mesh for physiological detection based on sweat. *JCIS Open* **2021**, *2*, 100007.

[3] Gao, C.; Yu, R.; Li, E.; Zhang, C.; Zou, Y.; Chen, H.; Lin, Z.; Guo, T. Adaptive immunomorphic hardware based on organic semiconductors and oxidized MXene heterostructures for feature information recognition. *Cell Rep. Phys. Sci.* **2022**, *3*, 100930.

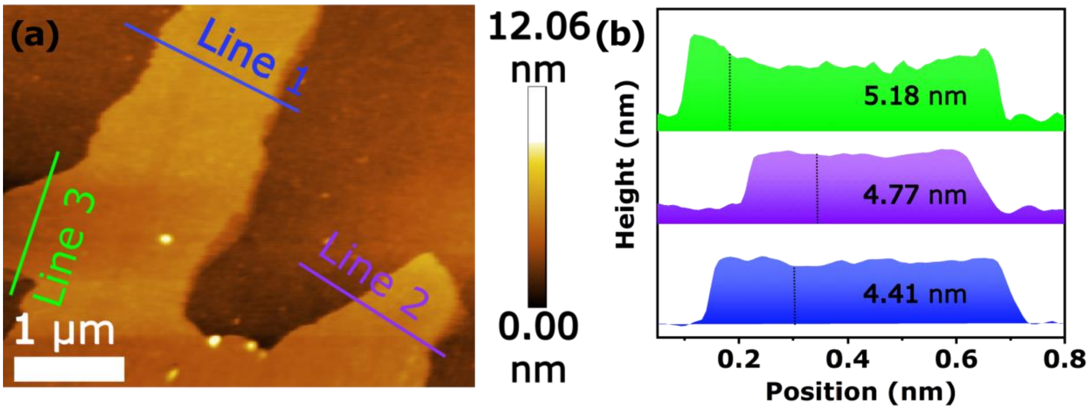

Figure S10. (**a**) AFM image and (**b**) height profiles of MT-600 °C.

The morphology information of MT-600 °C was further investigated by atomic force microscopy (AFM). As could be seen from the AFM image (Figure S10a and S10b), MT-600 °C showed a distinct layered structure. According to the AFM height profile, the average heights and thicknesses of MT-600 °C were measured to be 12.06 and 4.79 nm, respectively. The thickness of MXene in studies was generally about 2–4 nm [1]. However, the increase in the height of MT-600 °C indicated that the formation of TiO_2 nanoparticles in the interlayer structure leads to a larger interlayer spacing [2].

Reference

[1] Chen, J.; Zhao, Y.; Sun, M.; Liu, Z.; Liu, H.; Xiong, S.; Li, S.; Song, J.; Wang, K.Functionalized carbon fibers with MXene via electrochemistry aryl diazonium salt reaction to improve the interfacial properties of carbon fiber/epoxy composites. *J. Mater. Res. Technol.* **2022**, *19*, 3699–3712.

[2] Lukatskaya, R.; Mashtalir, O.; Ren, E.; Dall'Agnese, Y.; Rozier, P.; Taberna, L.; Naguib, M.; Simon, P.; Barsoum, W.; Gogotsi, Y. Cation Intercalation and High Volumetric Capacitance of Two-Dimensional Titanium Carbide. *Science* **2013**, *341*, 1502–1505.

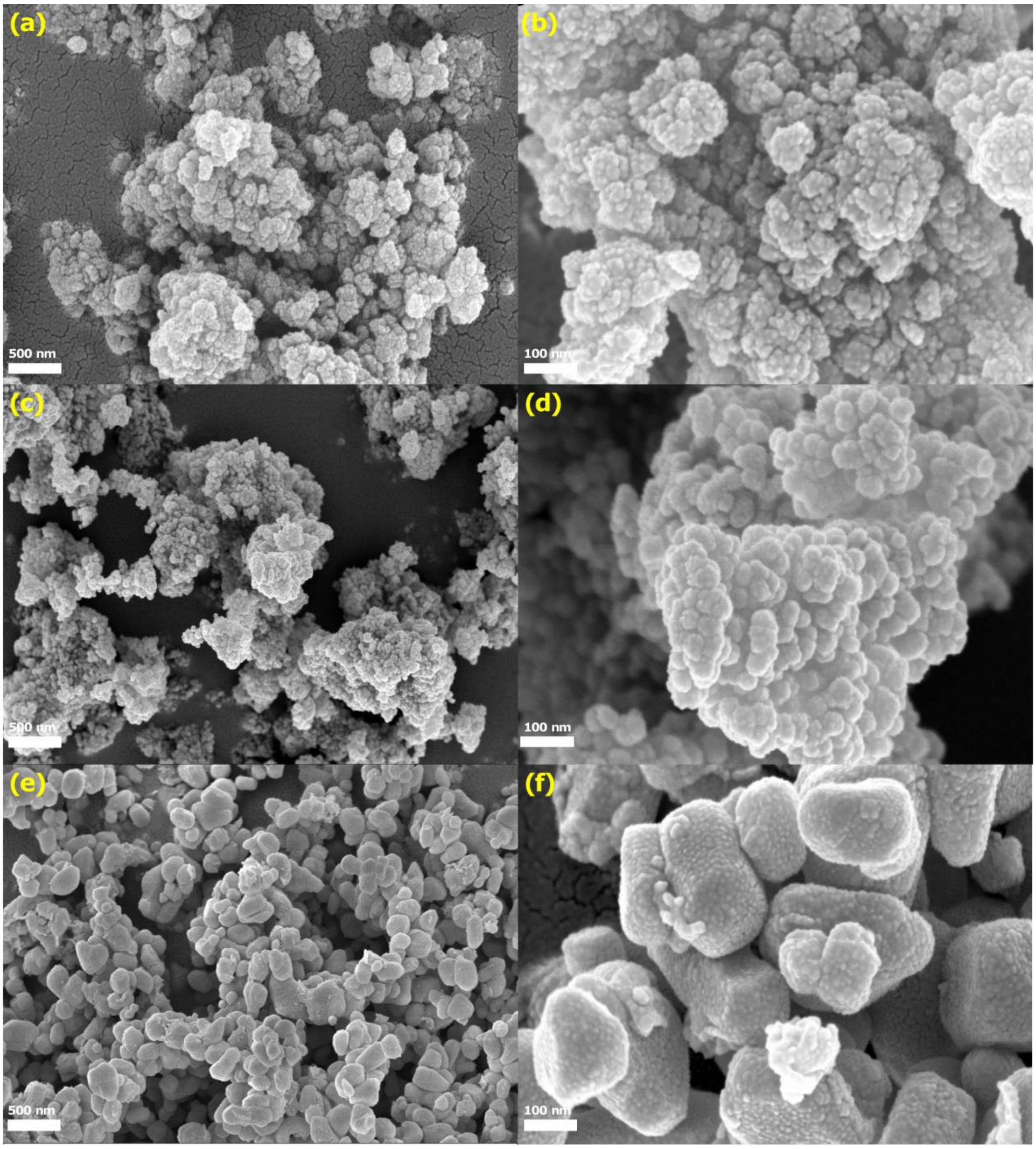

Figure S11. SEM images of (**a**)–(**b**) anatase TiO_2, (**c**)–(**d**) AR-750 °C, and (**e**)–(**f**) rutile TiO_2.

Moreover, as an experimental comparison, the detailed SEM images of different crystal structures of TiO_2 nanoparticles are shown in Figure S4. Figure S4 shows the SEM images of anatase, anatase/rutile, and rutile TiO_2 phases. Among them, Figures S11a-S11b show that the anatase phase TiO_2 sample was many agglomerated nanoparticles, with a narrow particle size distribution and poor dispersion. After calcination at 750 °C, as shown in Figure S11c-S11d, the particle size of the samples changed significantly. The agglomerated nanoparticles were further grown into secondary particles, which indicated that the TiO_2 sample gradually transformed from anatase phase to rutile phase, without melting. Figures S11e-S11f show that the grain size of the rutile phase TiO_2 sample increased significantly, which indicates that, at high temperature, due to the melting of the small anatase grained, the intergranular interface disappeared, specific surface area decreased, larger size of the TiO_2 was further formed, and no melting phenomenon occurred [1].

Reference

[1] Xu, H.; Wang, W.; Zhu, W.; Zhou, L.; Ruan, M. Hierarchical-Oriented Attachment: From One-Dimensional $Cu(OH)_2$ Nanowires to Two-Dimensional CuO Nanoleaves. *Cryst. Growth Des.* **2007**, *7*, 2720–2724.

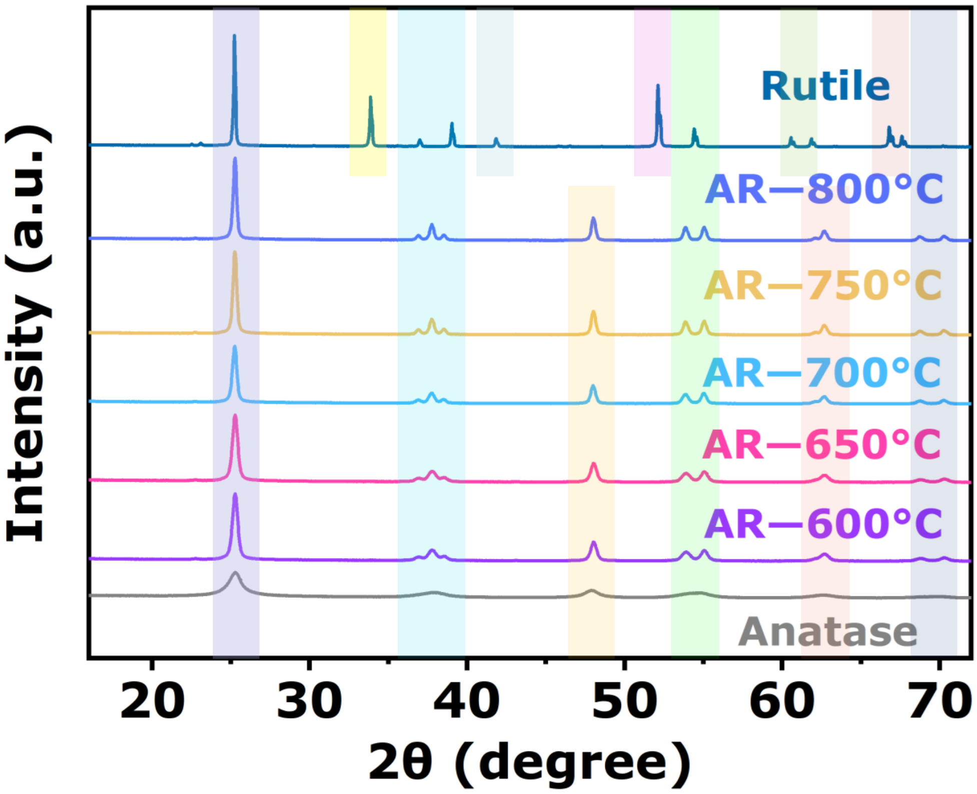

Figure S12. XRD patterns of anatase TiO_2, rutile TiO_2, and calcined anatase TiO_2 at different temperatures.

As a comparison, the XRD patterns of different crystal structures of TiO_2 nanoparticles were shown in Figure S12. Figure S12 shows that the diffraction peaks at 2θ = 25.3°, 37.8°, 48.5°, 54.2°, and 62.7° corresponded to (101), (004), (200), (105), and (204) planes in anatase TiO_2 phase (JCPDS PDF#21-1272) [1]. In addition, the diffraction peaks at 2θ = 27.4°, 36.3°, 41.2°, 44.1°, 54.2°, 56.6°, 62.7°, 64.0°, and 69.0° corresponded to (110), (101), (111), (210), (211), (220), (002), (310), and (301) planes in the rutile TiO_2 phase (JCPDS PDF#21-1276) [2]. Similarly, the existence of diffraction peaks of the respective anatase and rutile crystal forms could be seen from the calcined anatase TiO_2 at different temperatures.

Reference

[1] Zhang, X.; Chen, J.; Jiang, S.; Zhang, X.; Bi, F.; Yang, Y.; Wang, Y.; Wang, Z. Enhanced photocatalytic degradation of gaseous toluene and liquidus tetracycline by anatase/rutile titanium dioxide with heterophase junction derived from materials of Institut Lavoisier-125(Ti): Degradation pathway and mechanism studies. *J. Colloid Interface Sci.* **2020**, *588*, 122–137.

[2] Peng, C.; Wang, H.; Yu, H.; Peng, F. Active facets, interfacial charge transfer and Ti^{3+} doping for enhance photocatalytic activity. *Mater. Res. Bull.* **2017**, *89*, 16–25.

Phosphoprotein Sensing

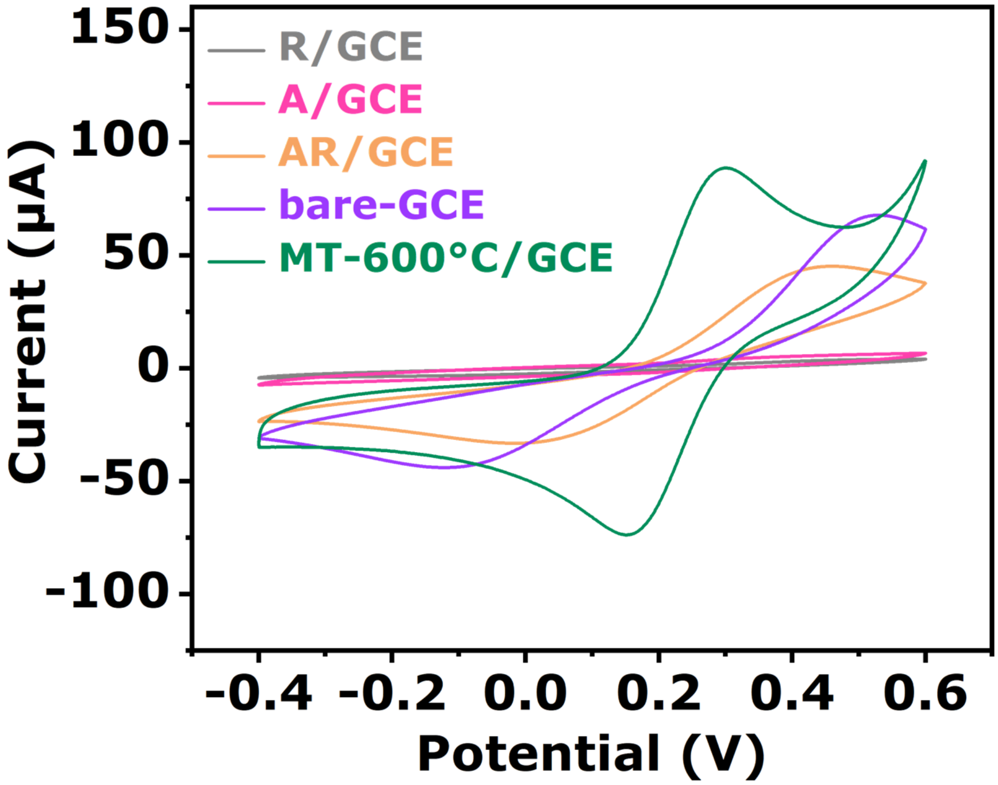

Figure S13. CV of the bare GCE, A/GCE, R/GCE, AR/GCE, and MT-600 °C/GCE in 5 mM $[Fe(CN)_6]^{3-/4-}$ solution containing 0.1 M KCl.

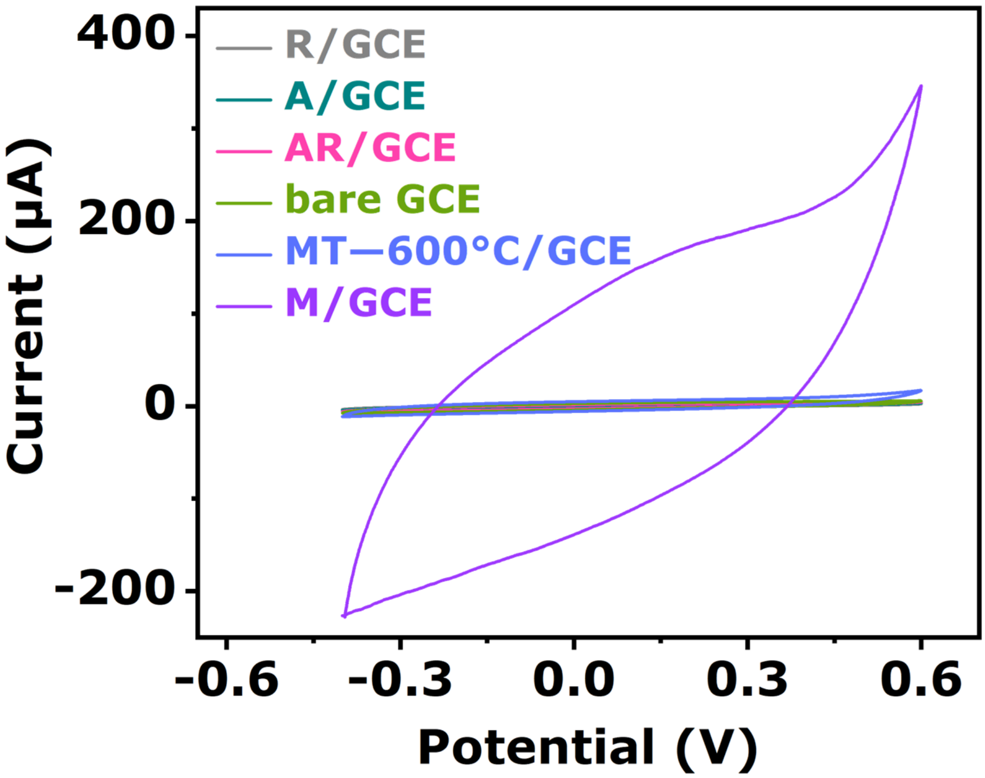

Figure S14. CV of the bare GCE, A/GCE, R/GCE, AR/GCE, M/GCE, and MT-600 °C/GCE in artificial sweat (pH = 7.0) containing 1 mg/mL β-Casein.

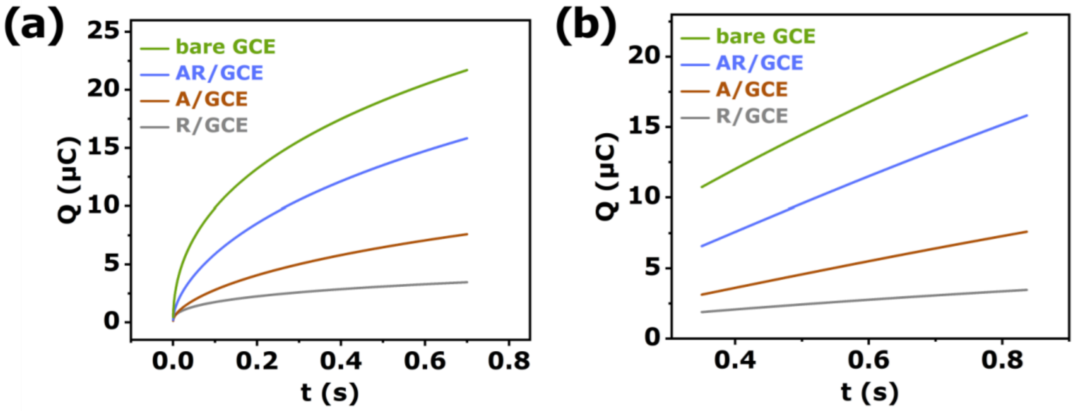

Figure S15. Plot of (**a**)Q-t curves and (**b**)Q-$t^{1/2}$ curves of the bare GCE, A/GCE, R/GCE, and AR/GCE in 0.1 mM $K_3[Fe(CN)_6]$ solution containing 1 M KCl.

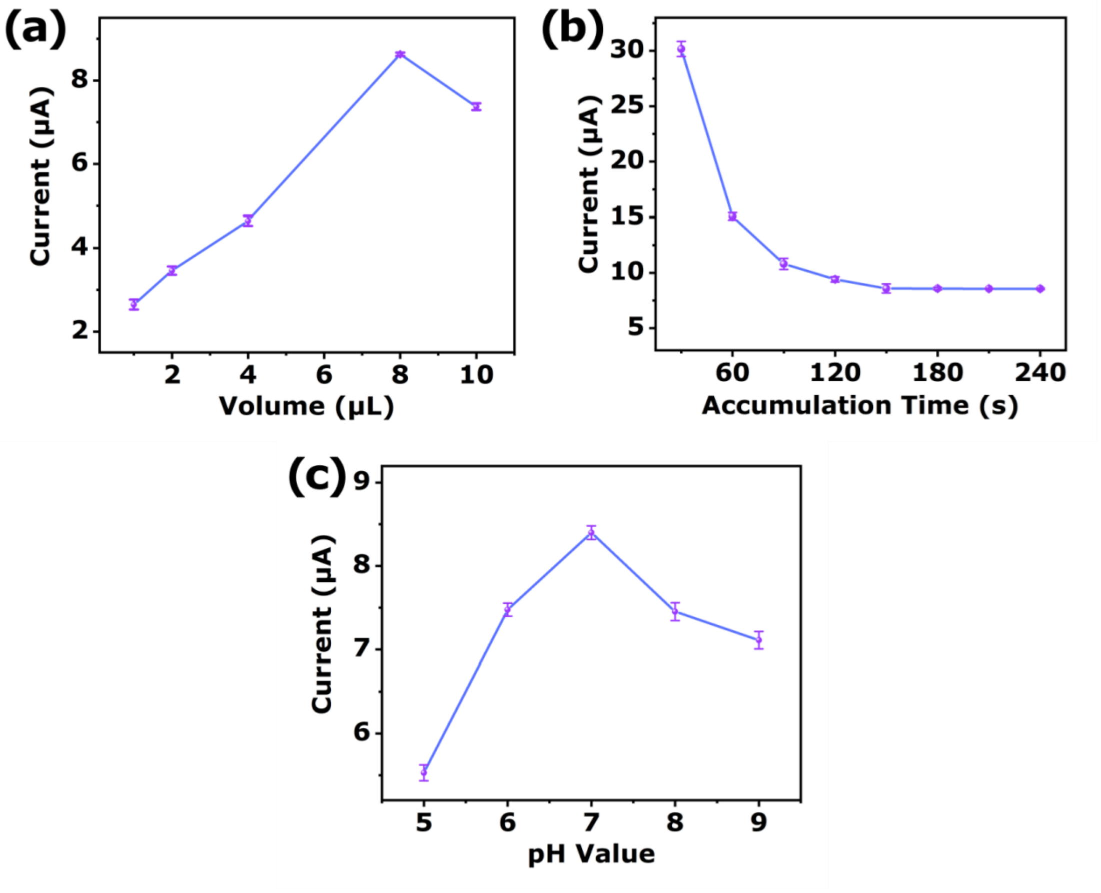

Figure S16. (**a**) Influence of the volume of MT-600 °C for the electrochemical response of β-Casein (1 mg/mL) in artificial sweat (pH = 7.0). (**b**) Influence of accumulation times on oxide peak currents of artificial sweat containing 1 mg/mL β-Casein (pH = 7.0). (**c**) Influence of the different pH values of MT-600 °C for the electrochemical response of β-Casein (1 mg/mL) in artificial sweat.

Table S1. The elemental analysis (C, O, F, and Ti atomic%) of the MXene and MT-600 °C performed by XPS.

Materials	C (Atomic%)	O (Atomic%)	F (Atomic%)	Ti (Atomic%)
MXene	49.14%	18.24%	14.70%	17.92%
MT-600 °C	30.90%	32.43%	19.83%	16.84%

Table S2. The C 1s elemental analysis composite state of the MXene and MT-600 °C performed by XPS.

Materials	Bond	Bingding Energy
MXene	COOH	288.9 eV
	C-O	286.23 eV
	C-C	284.8 eV
	C-Ti-O	282.53 eV
	C-Ti	281.7 eV
MT-600 °C	COOH	288.88 eV
	C-O	287.18 eV
	C-C	284.8 eV
	C-Ti-O	282.62 eV
	C-Ti	281.68 eV

Table S3. The O 1s elemental analysis composite state of the MXene and MT-600 °C performed by XPS.

Materials	Bond	Bingding Energy
MXene	C-OH	287.18 eV
	C-Ti-$(OH)_x$	531.13 eV
	Ti-O	533.18 eV
MT-600 °C	C-OH	288.88 eV
	C-Ti-$(OH)_x$	530.18 eV
	Ti-O	532.18 eV

Table S4. The Ti 2p elemental analysis composite state of the MXene and MT-600 °C performed by XPS.

Materials	Bonds	Bingding Energy
MXene	Ti_xO_y Ti $2p_{3/2}$	456.11 eV
	Ti-O Ti $2p_{3/2}$	457.42 eV
	Ti-F Ti $2p_{3/2}$	459.23 eV
	Ti-C Ti $2p_{3/2}$	455.06 eV
	Ti^{3+} Ti $2p_{1/2}$	463.04 eV
	Ti-O Ti $2p_{1/2}$	465.2 eV
	Ti-X Ti $2p_{1/2}$	455.02 eV
	Ti-C Ti $2p_{1/2}$	460.69 eV
MT-600 °C	Ti_xO_y Ti $2p_{3/2}$	456.72 eV
	Ti-O Ti $2p_{3/2}$	458.82 eV
	Ti-F Ti $2p_{3/2}$	458.17 eV
	Ti-C Ti $2p_{3/2}$	455.02 eV
	Ti^{3+} Ti $2p_{1/2}$	462.99 eV
	Ti-O Ti $2p_{1/2}$	464.4 eV
	Ti-X Ti $2p_{1/2}$	456.12 eV
	Ti-C Ti $2p_{1/2}$	460.6 eV

Table S5. BET physicochemical parameters of sensing materials.

Sensing Materials	S_{BET} (m^2/g)[a]	V_{Total} (cm^3/g)[b]	D (nm)[c]
Ti_3AlC_2	1.0892	0.007898	17.4197
MXene	2.3621	0.011941	23.9462
MT-200 °C	7.5112	0.040121	26.1093
MT-400 °C	8.2860	0.033183	24.8589
MT-600 °C	12.7380	0.042259	14.3820
MT-800 °C	21.2380	0.061318	11.5487

a BET specific surface; **b** total pore volume measured; **c** the pore diameter.

Table S6. The chemical bond change of β-Casein on MXene@anatase/rutile TiO_2 with different components.

Chemical Bonds in β-Casein	Bond Length (MXene@Anatase TiO_2/β-Casein)	Bond Length (MXene@Rutile TiO_2/β-Casein)	Bond Length (MXene@Anatase/Rutile TiO_2/β-Casein)
C-N	1.4573Å	1.4529Å	1.5092Å
C-C	1.5223Å	1.4970Å	1.5563Å
C-H	1.0994Å	1.0496Å	1.1089Å
C-O	1.4471Å	1.4251Å	1.4498Å
O-H	0.9992Å	0.9965Å	1.0035Å
N-H	1.0445Å	1.0437Å	1.0454Å
C-S	1.8731Å	1.84073Å	1.8876Å

Table S7. The chemical bond change of β-Casein on MXene@anatase TiO_2 with different components.

**Chemical Bonds in MXene@Anatase TiO_2**	**Bond Length (MXene@Anatase TiO_2/β-Casein)**
Ti-C	2.0337Å
Ti-O	1.9970Å

Table S8. The chemical bond change of β-Casein on MXene@rutile TiO_2 with different components

**Chemical Bonds in MXene@Rutile TiO_2**	**Bond Length (MXene@Rutile TiO_2/β-Casein)**
Ti-C	1.7905Å
Ti-O	1.9643Å

Table S9. The chemical bond change of β-Casein on MXene@anatase/rutile TiO_2 with different components.

**Chemical Bonds in MXene@Anatase/Rutile TiO_2**	**Bond Length (MXene@Anatase/Rutile TiO_2/β-Casein)**
Ti-C	2.0670Å
Ti-O	2.0153Å

Table S10. The adsorption energy for the different components of MXene@anatase/rutile TiO_2 on β-Casein

**Materials**	**Ads Energy (eV)**
MXene@anatase TiO_2	−1.33
MXene@rutile TiO_2	−0.99
MXene@anatase/rutile TiO_2	−2.26

Table S11. The diffusion coefficient for the different components of MXene@anatase/rutile TiO_2 on β-Casein.

**Materials**	**Diffusion Coefficient (m^2/s)**
MXene@anatase TiO_2	1.62×10^{-11}
MXene@rutile TiO_2	1.58×10^{-11}
MXene@anatase/rutile TiO_2	1.66×10^{-11}
